# Glycomic, Glycoproteomic, and Proteomic Profiling of Philippine Lung Cancer and Peritumoral Tissues: Case Series Study of Patients Stages I–III

**DOI:** 10.3390/cancers15051559

**Published:** 2023-03-02

**Authors:** Michael Russelle Alvarez, Qingwen Zhou, Jennyfer Tena, Mariana Barboza, Maurice Wong, Yixuan Xie, Carlito B. Lebrilla, Michelle Cabanatan, Ma. Teresa Barzaga, Nelia Tan-Liu, Francisco M. Heralde, Luster Serrano, Ruel C. Nacario, Gladys Cherisse Completo

**Affiliations:** 1 Department of Chemistry, University of California Davis, Davis, CA 95616, USA; 2 Institute of Chemistry, University of the Philippines Los Baños, Laguna 4031, Philippines; 3 Department of Anatomy, Physiology and Cell Biology, School of Veterinary Medicine, University of California Davis, Davis, CA 95616, USA; 4 Molecular Diagnostics and Cellular Therapeutics Laboratory, Lung Center of the Philippines, Quezon City 1100, Philippines; 5 College of Medicine, De La Salle Health Sciences Institute, Cavite 4114, Philippines; 6 College of Medicine, University of the Philippines Manila, Manila City 1000, Philippines

**Keywords:** Filipino, lung cancer, glycomics, proteomics, glycoproteomics

## Abstract

**Simple Summary:**

Protein glycosylation is a protein modification that contributes to a protein’s biological function. Over the years, it has been shown that protein glycosylation is correlated with cancer progression. Although much research has been performed on lung cancer protein glycosylation, clinical studies were conducted primarily on Caucasian populations. Hence, we are looking for protein glycosylation cancer biomarkers in a Philippine population, to identify which protein glycosylation modifications are unique to this population.

**Abstract:**

Lung cancer is the leading cause of cancer death and non-small cell lung carcinoma (NSCLC) accounting for majority of lung cancers. Thus, it is important to find potential biomarkers, such as glycans and glycoproteins, which can be used as diagnostic tools against NSCLC. Here, the N-glycome, proteome, and N-glycosylation distribution maps of tumor and peritumoral tissues of Filipino lung cancer patients (n = 5) were characterized. We present several case studies with varying stages of cancer development (I−III), mutation status (*EGFR, ALK*), and biomarker expression based on a three-gene panel (*CD133*, *KRT19*, and *MUC1*). Although the profiles of each patient were unique, specific trends arose that correlated with the role of aberrant glycosylation in cancer progression. Specifically, we observed a general increase in the relative abundance of high-mannose and sialofucosylated N-glycans in tumor samples. Analysis of the glycan distribution per glycosite revealed that these sialofucosylated N-glycans were specifically attached to glycoproteins involved in key cellular processes, including metabolism, cell adhesion, and regulatory pathways. Protein expression profiles showed significant enrichment of dysregulated proteins involved in metabolism, adhesion, cell−ECM interactions, and N-linked glycosylation, supporting the protein glycosylation results. The present case series study provides the first demonstration of a multi-platform mass-spectrometric analysis specifically for Filipino lung cancer patients.

## 1. Introduction

Despite advancements in cancer treatments, global cancer incidence, and mortality are still growing, reflecting several factors which include aging, population growth, cancer risk factors, and socioeconomic development. Of the estimated 19.3 million new cases and 10 million cancer deaths worldwide, 2,093,876 cases (11.6%) and 1,761,007 (18.4%) deaths are going to be attributed to lung cancer [1]. There are two main types of lung cancer—small cell lung cancer (SCLC) and more common non-small cell lung cancer (NSCLC), which account for approximately 85% of all lung cancer cases [2]. 

Risk factors that could contribute to the pathogenesis and development of NSCLC include environmental factors such as cigarette smoking [3,4] and alcohol consumption [5], as well as genetic factors such as *TP53* [6] and *EGFR* mutations [7]. The effect of race-ethnicity is becoming a factor as well, with different trends and cancer biomarkers being observed between different racial and ethnic backgrounds [8,9,10,11,12]. Thus, the differences in cancer physiology between populations need to be determined as well.

Protein glycosylation is one of the most complex and frequent modification involved in many cellular interactions such as host−pathogen interactions, cell differentiation and trafficking, and intra- and intercellular signaling [13]. Changes in cellular glycosylation patterns have been reported during cancer progression, and in particular, sialylation, fucosylation, O-glycan truncation, and N- and O-linked glycan branching are highly observed in tumor cells [14,15,16,17,18,19,20,21]. Several cancer-associated glycosylation changes have been documented in the two major types of protein glycosylation, that is, the addition of N- and O-linked glycans to glycoproteins, such as β1,6 branching, Sialyl Lewis antigens, α2,6-sialylated lactosamine, T, Tn, and sialyl-Tn antigens, and gangliosides/glycosphingolipids [14].

Previous studies show the association of increasing N-glycan structural complexity with cancer development, causing high functional variability in proteins, lipids, and metabolites [17,22,23,24,25]. Overexpression of glycan-processing enzymes is usually observed in cancer cells, resulting in enhanced expression of related glycan structures [24,26,27,28]. For example, in a primarily Caucasian cohort, the enzymes *Alpha1-6FucT, B4GALT2, MAN1A2*, and *MAN2A1* were overexpressed in lung cancer tissue samples [29]. Likewise, the abundance of high-mannose, fully galactosylated, and fucosylated N-glycans were also overexpressed in these lung cancer tissues [28]. In a separate Caucasian cohort (n = 100 cases, 199 controls for a discovery set; n = 108 cases, 216 controls for a test set), distinct glycans including Hex_4_HexNAc_5_Fuc_1_, Hex_5_HexNAc_6_Fuc_1_, NeuAc_2_, and Gal_4_ have been shown to provide a discriminating AUC (AUC = 0.74, 95% CI: 0.68−0.80) in a primarily Caucasian cohort [27]. In contrast, the N-glycan serum biomarkers from a Philippine lung cancer cohort (n = 26 patients, n = 22 age- and gender-matched) we have previously identified were Hex_6_HexNAc_5_NeuAc_1_, Hex_6_HexNAc_5_NeuAc_3_, Hex_6_HexNAc_5_Fuc_2_NeuAc_3_, Hex_7_HexNAc_6_Fuc_1_NeuAc_3_, Hex_4_HexNAc_3_NeuAc_1_, and Hex_5_HexNAc_4_Fuc_1_NeuAc_2_ [30].

Here, we report the cell surface N-glycome, proteome, and site-specific N-glycosylation distribution maps of a cohort (n = 5) of Filipino lung cancer patients’ tumor at different stages and peritumoral tissues using well-established mass spectrometry analyses.

## 2. Materials and Methods

### 2.1. Ethical Statement, Clinical Sample Collection, and Characterization

This research study was approved by the Lung Center of the Philippines Institutional and Ethics Review Board and was performed in accordance with the institutional guidelines/regulations (LCPIERB; ethics approval number: LCP-CS-003-2018); complete inclusion and exclusion criteria can be found in Table S1. Prior to recruitment, informed consent was obtained from all participants and/or their legal guardians. Patient eligibility included being Filipino citizens up to the second degree of consanguinity, with ages of 18−85 years old, and having neither tuberculosis nor HIV. Sources of malignant and nearby non-malignant tissues were from confirmed lung adenocarcinoma individuals who were chemotherapy- and radiotherapy-naïve. Malignant and non-malignant lung tissue samples were obtained directly through surgery (i.e., video-assisted thoracoscopic surgery (VATS), resection) at the Lung Center of the Philippines, Quezon City, Philippines. Tissue samples were collected through tissue biopsy or tissue resection/excision from patients diagnosed with lung adenocarcinoma. For case participants whose treatment involved resection/excision of their tumor tissues, tissue specimens consisting of tumor tissue and nearby normal tissue with approximately 2 cm^3^ in size and approximately 3−4 cm away were collected from the patients during their scheduled surgery. Clinically diagnosed lung cancer patients suggested for Thyroid transcription factor-1 (*TTF1*) confirmation had their specimen samples stored in 10% neutral buffered formalin and sent to the Histopathology Department, the Lung Center of the Philippines, Quezon City, Philippines for clinical profiling (ALK and EGFR mutation status; *CD133, KRT19*, and *MUC1* expression) using previously described RT-PCR methods [31]. Collected tissue samples were stored at −80 °C and transferred to the University of California Davis for mass spectrometric analyses.

### 2.2. Sample Preparation for Mass Spectrometric Analyses

Tissue samples were prepared according to previously established methods [32,33]. Briefly, cell surface membrane fractions were obtained, and the N-glycans, glycopeptides, and peptides were generated for mass spectrometric analyses. All sample preparation steps were performed on ice to keep the samples cold. Approximately 100 mg of each tissue sample was homogenized and washed twice with cold PBS buffer. Afterwards, 1.2 mL of HB buffer (0.25 M sucrose, 20 mM HEPES-KOH, 1:100 protease inhibitor cocktail) was added, and the samples were lysed using a sonicator probe. The samples were then centrifuged at 2000× *g* for 10 min at 4 °C. The pellet containing cell debris and nucleus was discarded, and the supernatant was subsequently centrifuged at 200,000× *g* for 45 min at 4 °C. The obtained pellet enriched in cell membrane proteins and glycoproteins were washed with 500 µL of 0.2 M Na_2_CO_3_ and centrifuged at 200,000× *g* for 45 min at 4 °C. Finally, to wash out excess salts and contaminants, the subsequent pellet was washed with deionized water for 200,000 g for 45 min (4 °C). The membrane fractions obtained were stored at −20 °C until further analysis.

### 2.3. N-Glycan Release and Glycomic Analysis Using Chip-QToF LC-MS/MS

N-glycans were released from the membrane fractions obtained by PNGase F treatment. The pellets obtained from the tissue sample preparation were first resolubilized in 100 µL N-glycan release solution (100 mM NH_4_HCO_3_ and 5 mM DTT) and then boiled for 1 min in a water bath. After cooling to room temperature, 2 µL PNGase F enzyme was added, and the samples were activated using a microwave (20 W) by heating at 33 °C for 10 min followed by incubation in a 37 °C water bath for 18 hours. N-glycans were purified using porous graphitized carbon solid-phase extraction (PGC-SPE) plates, eluted with 40% *v*/*v* acetonitrile and 0.05% trifluoroacetic acid in water, dried *in vacuo* and stored at −20 °C until analysis.

N-glycan sample preparation and mass spectrometry analysis was performed according to previously established methods [32,34]. N-glycan profiles were obtained using an Agilent nanochip-QTOF (quadrupole time-of-flight)-MS mass spectrometer. N-glycan samples were reconstituted in 40 μL of water, and 5 μL of the resulting solution was used for injection into the nano-LC-MS/MS system. Separation was performed using an Agilent PGC-Chip II with a 40 nL enrichment and 43 mm × 75 µm analytical column (particle size 5 µm) and a binary solvent system composed of mobile phase A (3% *v*/*v* acetonitrile and 0.1% *v*/*v* formic acid in water) and mobile phase B (90% *v*/*v* acetonitrile and 1% *v*/*v* formic acid in water). The gradient sequence for separation used was shown as follows: 0–2.5 min, 1% B; 2.5–20 min, 16% B; 20–35 min, 58% B; 35–40 min, 100% B 40–50 min, 100% B; 50.01–65 min, 0% B with a flow rate of 0.3 μL/min. Tandem MS spectra were acquired using collision-induced dissociation (CID), with spectra measured at 0.8 s per spectrum in the positive ion mode. 

Analysis of the N-glycan data was performed using MassHunter Qualitative Analysis Software B.07.00 (Agilent Technologies). N-glycans were identified using MassHunter’s Find by Molecular Feature algorithm, using previously defined parameters [32,34,35]. Matching of the monoisotopic masses obtained was performed against our in-house database for glycan composition identification and subsequently verified through their corresponding MS/MS spectra [36]. The relative abundance of each glycan in a sample was determined using the peak area of all glycans from extracted ion chromatograms. The N-glycans were subsequently classified using an in-house classification system: high-mannose, undecorated (with no fucose or sialic acid attached), fucosylated (with only fucose attached), sialylated (with only sialic acid attached), or sialofucosylated (with both fucose or sialic acid attached) [32,34,35,36]. The relative abundances of N-glycan groups (high-mannose, undecorated, fucosylated, sialylated, and sialofucosylated glycans) was calculated by adding the relative abundance of each individual glycan belonging to a specific glycan group. A further comparison of each N-glycan (according to the type and individual N-glycan species) was made using multiple t-tests (GraphPad Prism version 9.3.1 for Windows, GraphPad Software, San Diego, CA, USA, www.graphpad.com, accessed on 5 May 2022) at a significance level of ɑ = 0.05 after FDR correction. 

### 2.4. Proteomics and Glycoproteomic Analysis Using nLC-Orbitrap LC-MS/MS

For the proteomics and glycoproteomics analyses, membrane fractions were separately prepared from the tissue samples following the procedure above. The membrane proteins were reconstituted with 60 µL of 8 M urea and sonicated for 20 minutes for denaturation. Two microliters (2 µL) dithiothreitol (DTT, 550 mM in 50 mM NH_4_HCO_3_) was added to the samples, and the mixture was incubated for 50 min at 55 °C. The free cysteine was alkylated with 4 µL of iodoacetamide (450 mM) for 20 minutes in the dark at ambient temperature. The reaction was quenched by the addition of 420 µL buffer (50 mM NH_4_HCO_3_). Trypsin (10 µL, 0.1 mg/mL) was then added to the mixture, and tryptic digestion was performed at 37 °C for 18 h. For proteomic analysis, tryptic peptides were purified using C-18 SPE cartridges, eluted with 80% ACN and 0.1% TFA, dried in vacuo and stored at −20 °C before LC-MS/MS analysis. For site-specific glycoproteomic analysis, glycopeptides were enriched from tryptic digest preparation using HILIC solid-phase extraction, eluted with H_2_O containing 0.1% TFA, dried in vacuo and stored at −20 °C until LC-MS/MS analysis. Purified peptides and glycopeptides were quantified using a Pierce BCA assay kit following the manufacturer’s instructions (ThermoFisher, Waltham, MA, USA), and adjusted to concentrations of 0.5 µg/µL and 0.1 µg/µL, respectively, before injection in LC-MS/MS. 

Tryptic peptides and glycopeptides samples were analyzed in an UltiMate™ WPS-3000RS nanoLC system coupled with an Orbitrap Fusion Lumos MS system (ThermoFisher Scientific). One (1) microliter of each sample was injected, and the analytes were separated using an Acclaim™ PepMap™ 100C18 LC Column (75 µm × 150 mm, particle size: 2 µm; ThermoFisher Scientific) at a flow rate of 300 nL/min. Water containing 0.08% formic acid and 80% acetonitrile containing 0.1% formic acid were used as solvents A and B, respectively. MS spectra were collected with a mass range of *m*/*z* 700–2000 for MS1 and m/z of ≥120 for MS2, at a rate of 1.5 s per spectrum in the positive ionization mode. The filtered precursor ions in each MS spectrum were subjected to fragmentation through 30% higher-energy C-trap dissociation (HCD) using nitrogen gas as a carrier.

Mass spectrometry data were analyzed using the Byos workflow (Protein Metrics, Cupertino, CA, USA). For qualitative analysis using Byonic (Protein Metrics), proteins were identified against the human proteome database [37] using a precursor mass tolerance of 20 ppm and fragment mass tolerance of 10 ppm. Data analysis parameters used included C-terminal cleavage by trypsin (K and R cleavage sites) with at most two missed cleavages and the following peptide modifications: carbamidomethyl at the cysteine, oxidation at the methionine, deamidation at asparagine and glutamine, acetylation at the protein N-terminal, glutamine to pyro-glutamate at the N-terminal, and glutamate to pyro-glutamate at the N-terminal. Protein IDs were filtered at 1% FDR. For glycopeptide/glycoprotein identification, an additional search was performed in Byonic using an in-house N-glycan database. Each protein was quantified using Byologic (Protein Metrics) by quantifying the XIC (extracted ion chromatogram) area sum of the top 3 most abundant peptides per protein. The XICs were then normalized to total ion count before statistical analysis. On the other hand, glycoform quantification was normalized to each protein’s glycosite to yield the percentage glycan distribution of a particular glycoform. 

Statistical analysis (multiple *t*-tests with an FDR correction of 5%) was conducted using GraphPad Prism (version 9.3.1 for Windows, GraphPad Software, San Diego, CA, USA, www.graphpad.com, accessed on 5 May 2022) to identify significantly over- and under-expressed proteins, glycoproteins, and pathways. Pathway enrichment analysis were annotated using PantherGO [38] and then plotted as heatmaps in GraphPad Prism.

## 3. Results and Discussion

### 3.1. Clinical Profile of the Filipino Lung Cancer Cohort

As part of the cancer profiling and tumor characterization of the patient cases, three-gene expression analysis, EGFR, and ALK mutation analysis were performed on the samples collected from identified non-small cell lung cancer (NSCLC) Filipino patients at varying cancer stages (I-B, II-B, and III-A; Table 1). Four cases were analyzed for three marker gene-expression levels: CD133 (Prominin-1), KRT19 (Keratin 19), and MUC1 (Mucin 1). In the three-gene panel analysis, only one patient (FDT-01) had overexpressed CD133 and KRT19. All other cases had overexpression in only one of the genes: AF63-009 (MUC1), AM43-005 (CD133), and AM51-009 (CD133) (Table 1). In a previous study of Philippine patients, upregulation of at least two of the three marker genes was observed in 44% of Filipino NSCLC cases [39]. Upregulated MUC1 expression was also previously observed in NSCLC specimens, particularly in adenocarcinoma (86.3%) [40]. Kaplan−Meier survival curves showed lower overall survival (*p* = 0.011) and disease-free survival (*p* = 0.008), when MUC1 was overexpressed. In a meta-analysis of 11 studies involving 1004 NSCLC patients, CD133 overexpression was significantly correlated with a worse five-year overall survival (RR = 3.19, 95% CI: 2.05–4.98, *p* < 0.0001) [41]. Two cases (FDT-01 and AM53-021) tested positive for Epidermal Growth Factor Receptor (EGFR) mutations. FDT-01 had a mutation in Exon18, while AM53-021 had a mutation in Exon19 (Table 1). Such mutations could influence the patients’ response to tyrosine kinase inhibitor treatments, such as the NSCLC drug gefitinib. In a clinical study of NSCLC drugs gefitinib and erlotinib responses conducted on 166 patients (stage IIIB/IV lung adenocarcinoma) in Taiwan, patients harboring G719X mutations had a lower objective response rate (ORR = 36.8%) and disease control rate (DCR = 72.4%) than patients harboring Exon 19 deletions (ORR = 65.3%; DCR = 94.5%) and L858R mutations (ORR = 67.5%; DCR = 95.6%) [42]. 

### 3.2. N-Glycomic Profiles of the Filipino Lung Cancer Cohort

The potential of glycans and glycoproteins serving as biomarkers is based on the premise that these biomolecules are highly abundant, where as many as 50% of the proteins in the human body are glycosylated [43]. Glycoproteins play critical roles in cellular functions, signaling, differentiation, proliferation, and interactions. Interestingly, as broad as the functions of these biomolecules are in critical biological processes, aberrantly glycosylated proteins are highly associated with several human diseases, especially cancer [44].

**Figure 1:**
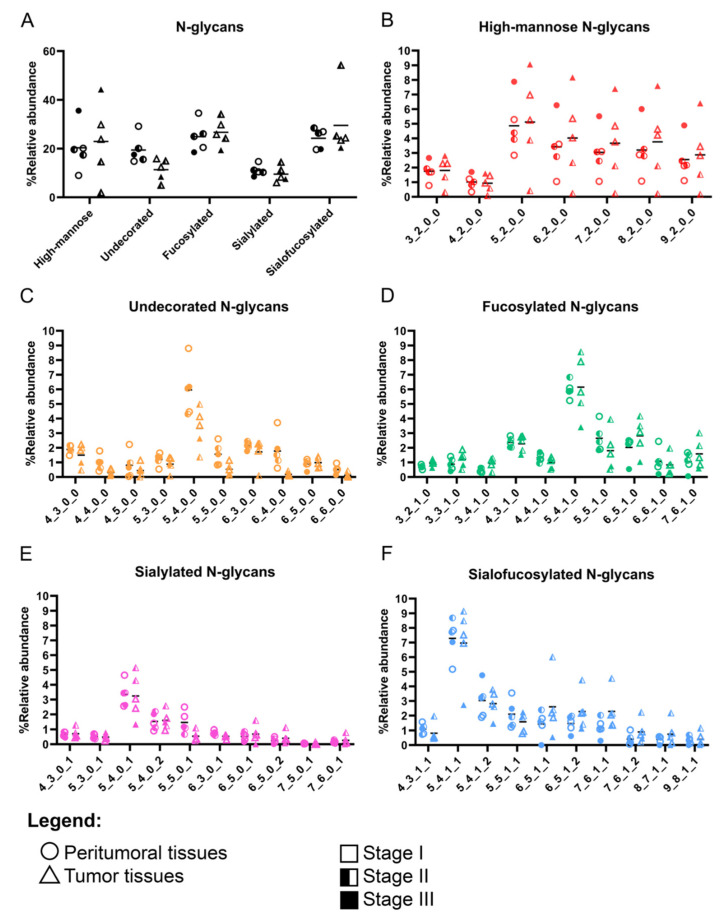
Cell surface N-glycome of Filipino tumor and peritumoral tissues categorized according to the lung cancer stage. (**A**) Relative abundances of high-mannose, undecorated, fucosylated, sialylated, and sialofucosylated N-glycans. Comparison of the relative abundances of the top 10 most abundant N-glycan per category—high-mannose (**B**), undecorated (**C**), fucosylated (**D**), sialylated (**E**), and sialofucosylated (**F**). N-glycan composition (*x*-axis) is provided in the format of hexose_N-acetylhexosamine_fucose_neuraminic acid.

Our results show that malignant tumor tissues generally had increased mannosylation, fucosylation, and complexity compared to their non-malignant peritumoral tissue neighbors (Figure 1, Figures S1−S5). More comprehensive quantification of the most abundant N-glycans per glycan type (Figure 1) showed a generally increased abundance in high-mannose N-glycans, fucosylation, and sialofucosylation, corresponding to a decreasing trend in undecorated and sialylated N-glycans (Figure 1). Furthermore, the comparison of the individual high-mannose N-glycan structures suggests that high-mannose N-glycans that were Hex_5_HexNAc_2_ up to Hex_2_HexNAc_9_ were more abundant, compared to those containing only the trimmed N-glycans (Hex_3_HexNAc_2_ and Hex_4_HexNAc_2_). Quantification of relative abundances of undecorated, fucosylated, and sialofucosylated N-glycans showed that the most abundant structures were the bi-antennary N-glycans Hex_5_HexNAc_4_, Hex_5_HexNAc_4_Fuc_1_, Hex_5_HexNAc_4_NeuAc_1_, and Hex_5_HexNAc_4_Fuc_1_NeuAc_1_, respectively. 

**Figure 2:**
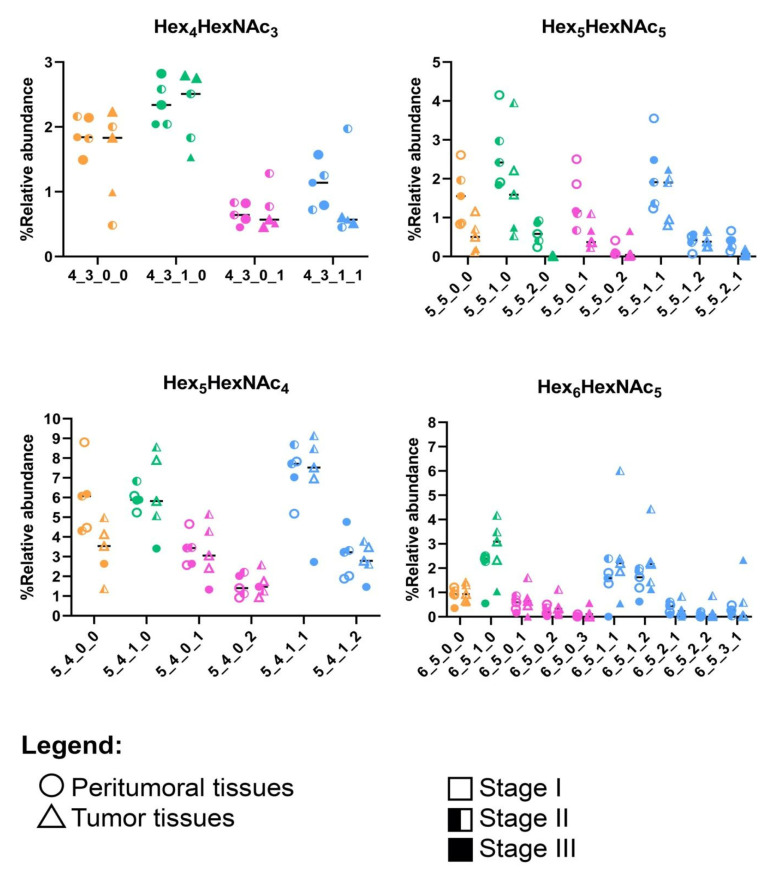
Specific comparison of the most abundant undecorated N-glycan structures and their decorations (e.g., fucosylation and sialylation): Hex_4_HexNAc_3_, Hex_5_HexNAc_4_, Hex_5_HexNAc_5_, and Hex_6_HexNAc_5_. N-glycans are color-coded according to the type: undecorated (orange), fucosylated (green), sialylated (pink), and sialofucosylated (blue). MS/MS fragmentation spectra can be found in Figure S7.

To further distinguish between the peritumoral and tumor tissues, we compared the decoration of the N-glycans with the highest abundances: Hex_4_HexNAc_3_, Hex_5_HexNAc_4_, Hex_5_HexNAc_5_, and Hex_6_HexNAc_5_ (Figure 2, Figure S6). Here, we consistently observed lower expression of the undecorated N-glycans in tumor tissues compared to that of peritumoral tissues. For Hex_4_HexNAc_3_, an increase in fucosylation (Hex_4_HexNAc_3_Fuc_1_) corresponded to a decrease in sialofucosylation (Hex_4_HexNAc_3_Fuc_1_NeuAc_1_). A decrease in the undecorated Hex_5_HexNAc_4_ corresponded to a subtle decrease in a doubly-sialylated glycan (Hex_5_HexNAc_4_NeuAc_2_). We also observed a decrease in the relative abundances of the undecorated N-glycan Hex_5_HexNAc_5_ and its fucosylated (Hex_5_HexNAc_5_Fuc_1_ and Hex_5_HexNAc_5_Fuc_2_), sialylated (Hex_5_HexNAc_5_NeuAc_1_ and Hex_5_HexNAc_5_NeuAc_2_), and sialofucosylated (Hex_5_HexNAc_5_Fuc_1_NeuAc_1_, Hex_5_HexNAc_5_Fuc_1_NeuAc_2_, and Hex_5_HexNAc_5_Fuc_2_NeuAc_2_) decorations, suggesting that the overall reduction in expression of Hex_5_HexNAc_5_ N-glycans corresponded to a reduction in its decoration as well. We also observed a decrease in the relative abundance of Hex_6_HexNAc_5_, a tri-antennary N-glycan, in both peritumoral and tumor samples, compared to the other undecorated N-glycans. Interestingly, the corresponding fucosylated (Hex_6_HexNAc_5_Fuc_1_) and sialofucosylated (Hex_6_HexNAc_5_Fuc_1_NeuAc_1_ and Hex_6_HexNAc_5_Fuc_1_NeuAc_2_) decorated N-glycans were observed to be higher in tumor samples compared to peritumoral tissues.

It was interesting to see that the glycosylation patterns of the Philippine lung cancer cohort were in agreement with prior reports on glycosylation profiles of lung cancer [27,28,30]. Similarly, increased high-mannose N-glycans have been observed in both formalin-fixed paraffin-embedded (FFPE) tissues [45] and in fresh lung cancer tissues [28]. This observation has been previously linked with downregulation of mannosidase genes in cancer and is thought to possibly contribute to cancer pathogenesis [26,29]. Another observation in our study is the overexpression of fucosylated and sialofucosylated N-glycans (Figure 1 and Figure 2). Core- and antennary-fucosylation and the formation of neutral and sialylated Lewis antigens have been reported to contribute to cancer progression [14,16]. In particular, FUT8 (alpha-1,6-fucosyltransferase), the only enzyme that participates in the addition of fucose residues to the core (core-fucosylation), was previously reported to be upregulated in lung cancer [46].

### 3.3. Proteomic Profiles of the Filipino Lung Cancer Cohort

**Figure 3:**
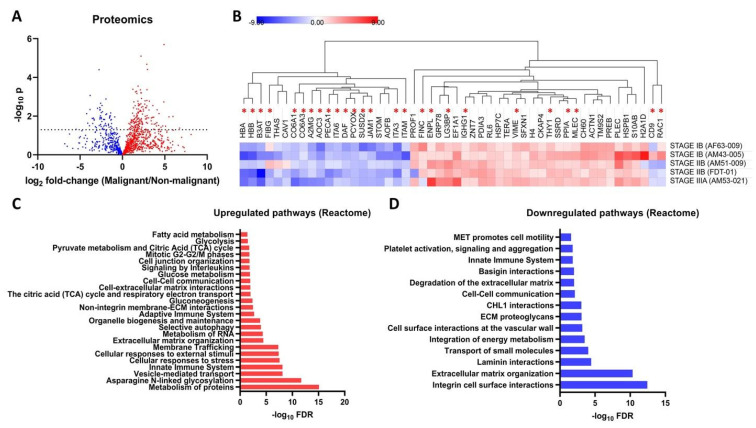
Cell surface proteome of tumor and peritumoral tissues from Filipino lung cancer patients. (**A**) Volcano plot showing differentially expressed proteins (222 significantly different proteins) between malignant (tumor)/non-malignant (peritumoral) tissue. (**B**) Heatmap of selected highly-significantly different proteins between tumor and peritumoral tissue. Values are expressed as log_2_ fold-change tumor/peritumoral tissue. The heatmap rows are labeled according to the patient source. Proteins with reported glycosylation sites are labeled with asterisks. Selected significant Upregulated (**C**) and downregulated (**D**) Reactome pathways (FDR = 5%) are annotated using PantherDB.

Label-free quantitative proteomics was performed to identify changes in protein expression profiles in lung cancer tumor tissues compared to the corresponding peritumoral tissues. Here, 1577 proteins were detected, quantified and used for a comparison between the two groups, with 222 proteins found to be significantly different between the groups (Figure 3A,B). Out of the 222 significantly different proteins, 157 were overexpressed, and 65 were underexpressed in tumor samples compared to in peritumoral samples. Interestingly, the majority of the proteins that were consistently overexpressed and underexpressed in tumor compared to in peritumoral tissues were glycosylated (Figure 3B). Significantly overexpressed proteins were mapped into 148 Reactome pathways (Figure 3C, Figure S9, Table S2), which included N-linked glycosylation (q = 2.04 × 10^−12^), extracellular matrix organization (q = 3.68 × 10^−5^), glucose metabolism (q = 1.33 × 10^−2^), autophagy (q = 1.07 × 10^−4^), extracellular matrix interactions (q = 1.13 × 10^−2^), mitotic phases (q = 1.71 × 10^−2^), and glycolysis (q = 3.45 × 10^−2^). On the other hand, significantly underexpressed proteins were mapped into 35 Reactome pathways (Figure 3D, Figure S10, Table S3), which included cell−cell communication (q = 7.61 × 10^−3^), cell motility (q = 2.64 × 10^−2^), and integrin cell surface interactions (q = 3.74 × 10^−13^). 

Asterisks indicate glycoproteins. These overexpressed pathways are specifically relevant to our study due to the involvement of certain glycoproteins and glycosylation genes. For example, the overexpressed proteins COPA (coatomer subunit alpha), SE1L1 (protein sel-1 homolog-1), TMEDA (transmembrane emp24 domain-containing protein 10), GANAB (neutral alpha-glucosidase AB), PREB (prolactin regulatory element-binding protein), COPB (coatomer subunit beta), TERA (transitional endoplasmic reticulum ATPase), ACTZ (alpha-centractin), SNAA (alpha-soluble NSF attachment protein), TBB4B (tubulin beta-4B chain), RPN2 (dolichyl-diphosphooligosaccharide--protein glycosyltransferase subunit 2), ARF4 (ADP-ribosylation factor 4), PDIA3 (protein disulfide-isomerase A3), OST48 (dolichyl-diphosphooligosaccharide--protein glycosyltransferase 48 kDa subunit), NSF (vesicle-fusing ATPase), CALX (calnexin), LMAN2 (vesicular integral-membrane protein VIP36), TBA1A (tubulin alpha-1A chain), TMED9 (transmembrane emp24 domain-containing protein 9), TBA1B (tubulin alpha-1A chain), MLEC (malectin), and RPN1 (dolichyl-diphosphooligosaccharide--protein glycosyltransferase subunit 1) were found to be involved in the N-linked glycosylation pathway [47]. In addition, several overexpressed proteins are also involved in several sub-pathways linked to the N-linked glycosylation pathway. COPA, TMEDA, PREB, COP, ACTZ, SNAA, TBB4B, ARF4, NSF, LMAN2, TBA1A, TMED9, and TBA1B are also involved in ER to Golgi transport. Several are enzymes directly involved in the biosynthesis of glycans, such as RPN1, RPN2, and OST48 (OST complex), while CALX and PDIA3 are involved in the unfolded protein response [47].

Interestingly, there are some pathways that are enriched in both over- and underexpressed proteins, particularly in cellular interactions with the extracellular matrix. The extracellular matrix organization and interaction pathways involved the overexpressed proteins ACTB (actin, cytoplasmic 1), ACTN1 (alpha-actinin-1), CTND1 (catenin delta-1), FLNA (filamin-A), PLEC (plectin), LAMC1 (laminin subunit gamma-1), ITAV (integrin alpha-V), ITB5 (integrin beta-5), and TKT (transketolase) [48,49]. The laminin (q = 3.63 × 10^−5^), integrin, and basigin (q = 1.00 × 10^−2^) interactions were mediated by underexpressed proteins ITA2 (integrin alpha-2), ITB1 (integrin beta-1), ITA1 (integrin alpha-1), ITA3 (integrin alpha-3), ITA6 (integrin alpha-6), PECA1 (platelet endothelial cell adhesion molecule), CO6A3 (collagen alpha-3(VI) chain), JAM1 (junctional adhesion molecule A), ICAM1 (intercellular adhesion molecule 1), CO6A1 (collagen alpha-1(VI) chain), ITAM (integrin alpha-M), and ESAM (endothelial cell-selective adhesion molecule) [50,51]. Other studies identified protein signatures of cancer from clinical tissue samples [52]. Tumor cells generally present dysregulated expression of proteins involved in the 10 hallmarks of cancer [53,54]. 

### 3.4. Glycoproteomic Profiles of the Filipino Lung Cancer Cohort

**Figure 4:**
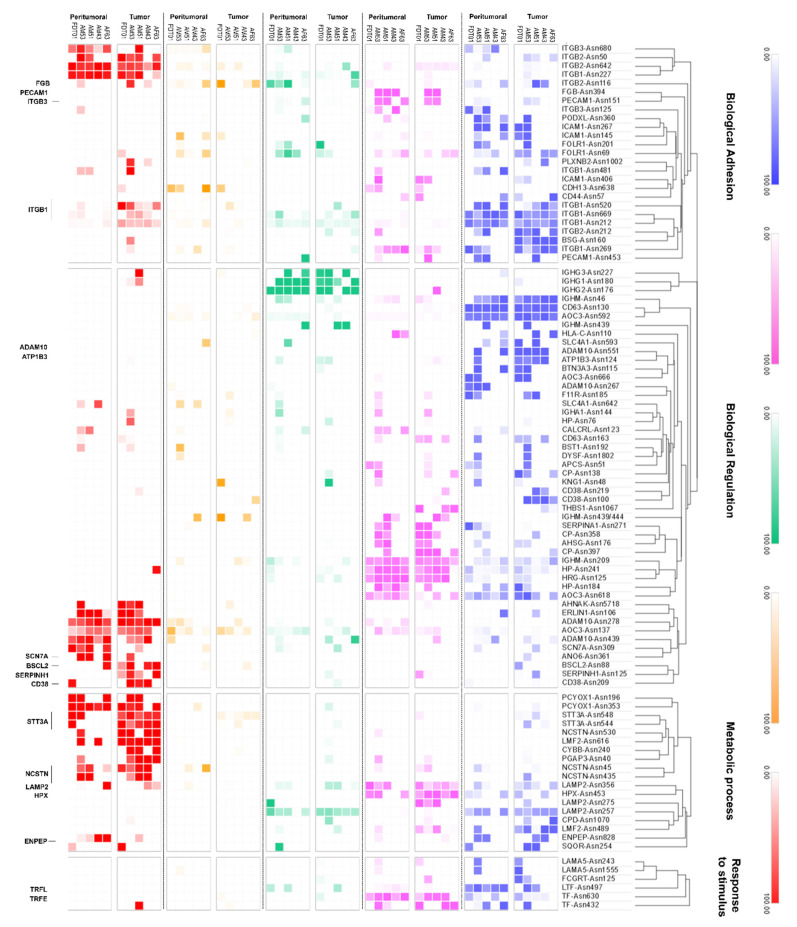
Glycan distribution maps of N-glycospeptides derived from cell surface proteins from tumor and peritumoral tissues, categorized according to the type—high-mannose (red), undecorated (orange), fucosylated (green), sialylated (pink), and sialofucosylated (blue)—and annotated according to biological processes—biological adhesion, regulation, metabolic process, and response to stimulus—as characterized using site-specific glycoproteomics.

To further characterize and understand the differences in protein glycosylation between the tumor and peritumoral samples of Filipino lung cancer patients, we conducted a site-specific glycopeptide analysis. We identified 492 glycoproteins, spanning 1656 specific asparagine glycosites, and 7389 unique glycopeptides. The glycan distribution for each protein glycosite was calculated by summing the relative abundance of each N-glycan type—high-mannose, undecorated, fucosylated, sialylated, and sialofucosylated. Glycoproteins were further annotated based on biological processes using PantherGO [38], to yield an annotated lung tissue protein glycosylation map with interesting and novel results (Figure 4). Firstly, we observed that glycoproteins tended to have low levels of undecorated glycosylation, which was correlated to our N-glycomics data (Figure 1). Indeed, we observed that high-mannose and sialofucosylated glycosylation were highly abundant in both N-glycomics and site-specific glycoproteomics data. After annotating the glycoproteins with PantherGO [38], we found that the glycoproteins with a high abundance of high-mannose glycosylation were mostly involved in Biological Adhesion, Regulation, and Metabolic Processes (Figure 4). Specifically, we noted a consistent increase (in tumor tissue compared to in peritumoral tissue) in high-mannose abundance in three glycosylation sites of ITGB1 (integrin beta-1, P05106): Asn212, Asn520, and Asn669. The other glycoproteins involved in Biological Adhesion that had a high abundance of high-mannose glycosylation (in tumor tissue compared to in peritumoral tissue) were ITGB2 (P05107) and ITGB3 (P05106), suggesting the relevance of high-mannose glycans in the function of beta-family integrins. For Biological Regulation, we consistently observed a higher abundance of high-mannose glycosylation (in tumor tissue compared to in peritumoral tissue) in glycoproteins such as BSCL2-Asn88 (Q96G97), SERPINH1-Asn125 (P50454), and CD38-Asn209 (P28907). However, for Metabolic Process, we observed a higher abundance of high-mannose glycosylation (in tumor tissue compared to in peritumoral tissue) in several glycosites of STT3A (P46977): Asn544 and Asn548. Interestingly, STT3A was involved in N-glycan biosynthesis as part of the oligosaccharyltransferase (OST) complex [55]. 

A high abundance of sialofucosylation was observed in glycoproteins involved in Adhesion, Regulation, and Metabolism (Figure 4). For the glycoproteins, annotated to be involved in Biological Regulation, ADAM10-Asn124 (O14672) were found to have increased sialofucosylation in tumor tissues compared to in peritumoral tissues. Specifically, ADAM10 is an endopeptidase that regulates the activity of various cell-membrane proteins, such as TspanC8 [56], APP [57], heparin-binding epidermal growth factor [58], and L1CAM [59]. The proteins LAMP2-Asn356 (P13473) and HPX-Asn453 (P02790), involved in metabolic processes, were found to have a consistently higher abundance of sialofucosylated glycans in tumor tissue compared to in peritumoral tissue. Additionally, several glycoproteins identified were integrins (ITB1/2/3/5/6), growth factor receptors (EGFR and INSR), and immunoglobulins (IGHA1/2, IGHG1/2/3/4, and IGHM). These glycoproteins have been implicated in cancer progression, such as the synergistic roles of integrin and growth factor signaling [60,61]. Furthermore, tumor cells also secrete cancer-derived immunoglobulins that play roles in cancer survival [62,63].

An increase in fucosylation in tumor tissues has also been reported in cancer studies. High expression of FUT8, the enzyme responsible for synthesizing core fucosylated N-glycans, is correlated to poor prognosis of most patients diagnosed with non-small cell lung carcinoma [64]. On the other hand, an increased sialofucosylation is one of the main characteristics of malignant transformation [14,16], where the increase in decoration, either with fucose or sialic acid residues, of N-glycan types has been correlated to enhanced glycosyltransferase activity, decrease in sialidase and fucosidase activity, or increased glycoprotein production. Determining the concentration of sialylated and fucosylated glycoprotein has proved helpful in evaluating the clinical utility of these glycan types for the diagnosis of non-small cell lung cancer [65]. 

Interestingly, site-specific glycoproteomics showed apparently higher representation of sialylation of glycoproteins (Figure 4), in contrast to N-glycomics results (Figure 1). The glycoproteins with a high abundance of sialylation—HPT-Asn241 (P00738), HRG-Asn125 (P04196), AOC3-Asn618 (Q16853), LAMP2-Asn356 (P13473), TRFE-Asn630, and -Asn432 (P02787)—had relatively low abundances in our proteomics quantification. Our results suggest the preferential sialylation of these specific glycosites, even at relatively lower protein expression. Indeed, we observed primarily sialylated and sialofucosylated glycans attached to these sites in our site-specific glycoproteomic analysis. In a previous biomarker study (n = 25 patients, 21 healthy volunteers) of Philippine lung cancer sera, we similarly observed significantly increased abundances of sialofucosylated immunoglobulin M-Asn46 and Asn 209, serotransferrin-Asn630, and Alpha-1-antitrypsin-Asn107 and Asn271 glycoproteins [30]. 

**Figure 5:**
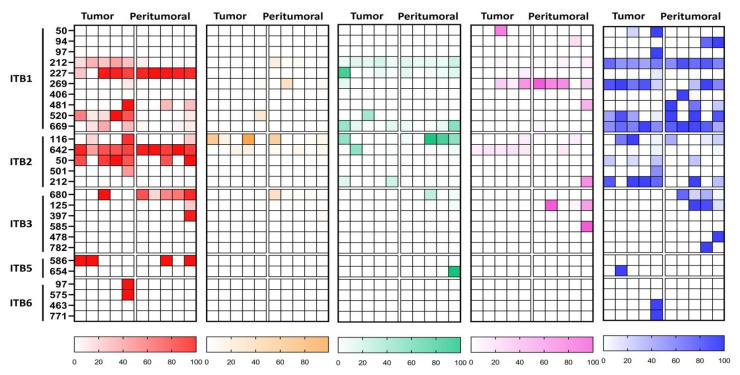
Site-specific N-glycosylation of selected integrin glycoproteins—ITB1, ITB2, ITB3, ITB5, and ITB6—compared between tumor and peritumoral tissues and categorized according to the type.

Due to the consistent overexpression of high-mannose glycans in integrin proteins, we decided to focus on the beta-integrins. Integrins are the protein family of interest in our study, particularly due to their relevance in various biological processes and their implication in cancer pathways [60,61,66,67]. Glycosylation plays a crucial role in integrin function, with implications in cancer progression, such as in heterodimerization, stabilization, and ligand interactions [68,69,70,71]. Particularly, the aberrant decoration of sialic acid and fucose to integrin N-glycans has been linked to tumor behavior, such as enhanced metastatic potential [69,70,71,72]. In our study, we observed that changes in glycosylation of the integrins ITB1, ITB2, ITB3, ITB5, and ITB6 correlated well with the glycomics results, that is, overexpression of high-mannose and sialofucosylated glycans (Figure 4 and Figure 5). For example, in ITB1, we consistently observed an increased relative abundance of high-mannose glycans at glycosites Asn212, 520, and Asn669, a decrease in relative abundance of sialylated glycans at glycosite Asn269, and a loss of sialofucosylation at glycosites Asn481 and Asn520 (Figures S11−S15). In ITB2, we observed increased relative abundances of sialylated glycans at Asn642 and sialofucosylated glycans at glycosite Asn212.

## 4. Conclusions

In conclusion, we used the state-of-the-art mass spectrometry platforms to characterize the cell surface N-glycome, proteome, and glycan distribution maps of a cohort of Filipino lung cancer samples (n = 5), who were previously characterized clinically for *ALK* and *EGFR* mutations. We compared the glycosylation between tumor and peritumoral tissues originating from the same patient and found that there was overexpression of high-mannose and sialofucosylated glycans similar to those in the reported literature. Using site-specific glycoproteomics, these N-glycans were attributed to glycoproteins involved in cancer hallmark pathways such as adhesion, regulation, and metabolism. In particular, we noted the relevance of this change in protein glycosylation for a set of integrin proteins *ITB1, ITB2, ITB3, ITB5*, and *ITB6*, which are known to be implicated in cancer. Finally, quantitative proteomics results rounded out our findings by correlating changes in glycosylation with upregulation and downregulation of relevant cancer-associated pathways, such as in metabolism, cell adhesion, regulation, and cell signaling pathways. To date, this is the first demonstration of a multi-platform mass-spectrometric analysis of N-glycome, proteome, and glycoproteome, in a case series study of lung cancer in the Philippine population. It will be interesting to observe similar changes in cancer stages with a significantly larger sample size, and, as such, we plan to collect and analyze more samples for follow-up and validation of the results obtained in a future study.

## Figures and Tables

**Table 1:** Demographic and genetic profiles of the five Filipino lung cancer patients in the cohort.

Subject ID	Stage	Age/Sex	Mutation Status	3-Gene Panel Expression				
			*TTF1*	*ALK*	*EGFR*	*CD133*	*KRT19*	*MUC1*
AF63-009	I-B	63/F	Positive	Negative	Negative	Down	Down	Up
AM43-005	I-B	43/M	Positive	Negative	Negative	Up	Down	Down
AM51-009	II-B	51/M	Positive	Negative	Negative	Up	Down	Down
FDT-01	II-B	75/F	Positive	Negative	Positive (Exon18)	Up	Up	Down
AM53-021	III-A	53/M	Positive	Negative	Positive (Exon19)	No data	No data	No data

## Data Availability

The datasets generated and analyzed during the current study are available on the MassIVE data repository: glycomics (doi:10.25345/C5Q27C, MSV000088273), proteomics (doi:10.25345/C5985Z, MSV000088193), and glycoproteomics (doi:10.25345/C5S86P, MSV000088197).

## Supplementary Materials

The following supporting information can be downloaded at: https://www.mdpi.com/article/10.3390/cancers15051559/s1, Figure S1. Representative XICs of N-glycome in tumor and peritumoral tissue from patient AF63-009; Figure S2. Representative XICs of N-glycome in tumor and peritumoral tissue from patient AM53-021; Figure S3. Representative XICs of N-glycome in tumor and peritumoral tissue from patient FDT-01; Figure S4. Representative XICs of N-glycome in tumor and peritumoral tissue from patient AM51-009; Figure S5. Representative XICs of N-glycome in tumor and peritumoral tissue from patient AM43-005; Figure S6. Relative abundances of all N-glycans across all samples; Figure S7. Representative MS/MS spectra of selected N-glycan structures detected in tissue samples; Figure S8. Representative MS/MS spectra of ITGB1 peptides detected in tissue samples; Figure S9. Significantly enriched Reactome pathways based on significantly overexpressed proteins in tumor tissues; Figure S10. Significantly enriched Reactome pathways based on significantly underexpressed proteins in tumor tissues; Figure S11. Representative MS/MS spectra of ITGB1 glycopeptides at glycosite Asn212 detected in tissue samples; Figure S12. Representative MS/MS spectra of ITGB1 glycopeptides at glycosite Asn269 detected in tissue samples; Figure S13. Representative MS/MS spectra of ITGB1 glycopeptides at glycosite Asn481 detected in tissue samples; Figure S14. Representative MS/MS spectra of ITGB1 glycopeptides at glycosite Asn520 detected in tissue samples; Figure S15. Representative MS/MS spectra of ITGB1 glycopeptides at glycosite Asn669 detected in tissue samples; Table S1. Inclusion and exclusion criteria; Table S2. Reactome pathways mapped by significantly overexpressed proteins; Table S3. Reactome pathways mapped by significantly underexpressed proteins.
