# Supplementary material for: Glycomic, Glycoproteomic, and Proteomic Profiling of Philippine Lung Cancer and Peritumoral Tissues: Case Series Study of Patients Stages I–III
Source: Cancers (Basel). 2023 Mar 2;15(5):1559. doi: 10.3390/cancers15051559 (PMC10001221; doi:10.3390/cancers15051559)
Supplement: Supplementary file 1 [file cancers-15-01559-s001.zip › cancers-2031539-supplementary.pdf]

cancers

MDPI

Glycomic, Glycoproteomic, and Proteomic Profiling of Philippine Lung Cancer and Peritumoral Tissues: Case series study of patients Stages I–III

Michael Russelle Alvarez , Qingwen Zhou , Jennyfer Tena , Mariana Barboza , Maurice Wong , Yixuan Xie , Carlito B. Lebrilla , Michelle Cabanatan , Ma. Teresa Barzaga , Nelia Tan-Liu , Francisco M. Heralde III , Luster Serrano , Ruel C. Nacario and Gladys Cherisse Completo

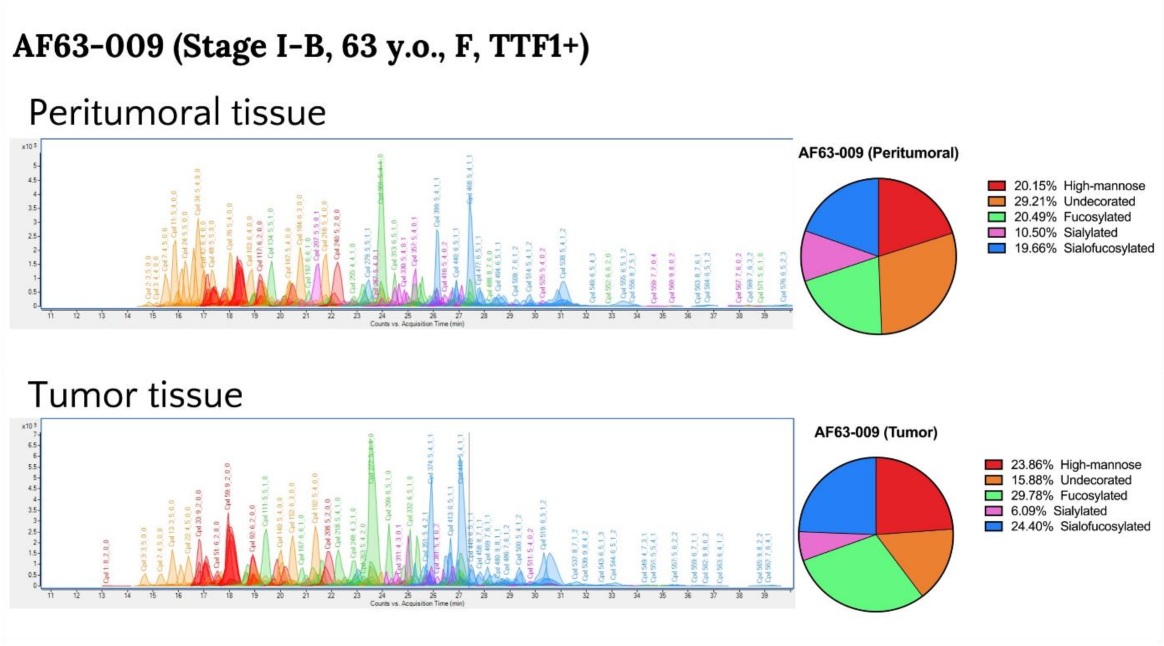

Figure S1. Representative XICs of tumor and peritumoral tissue N-glycome from patient AF63-009.

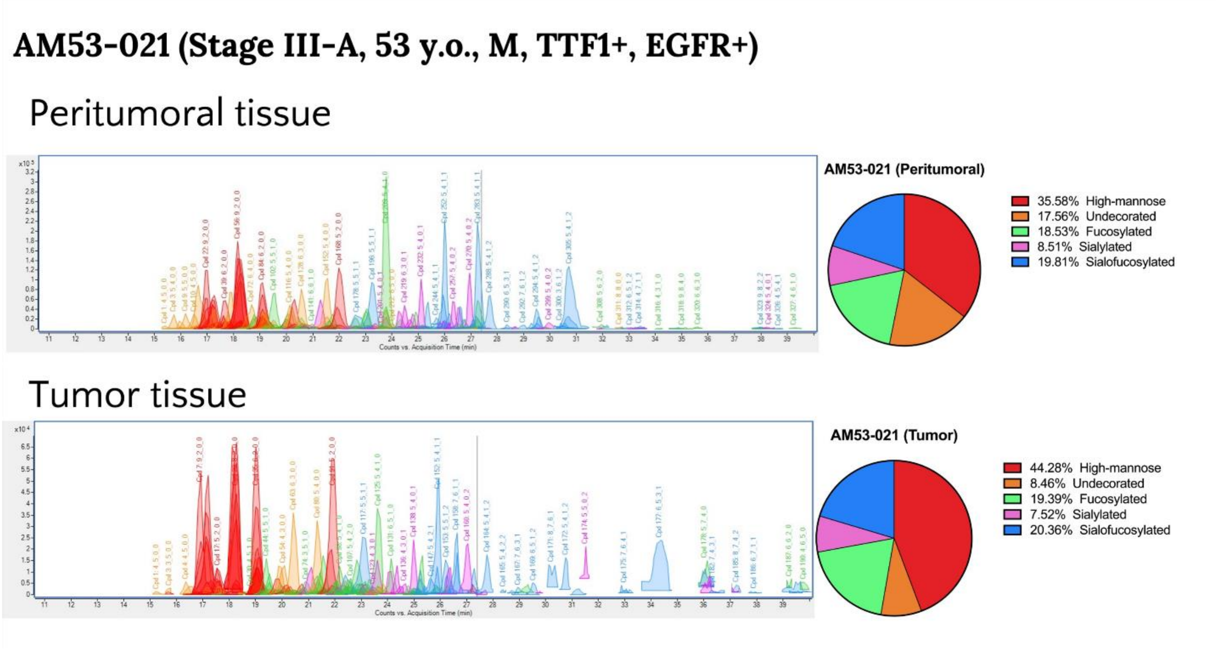

Figure S2. Representative XICs of tumor and peritumoral tissue N-glycome from patient AM53-021.

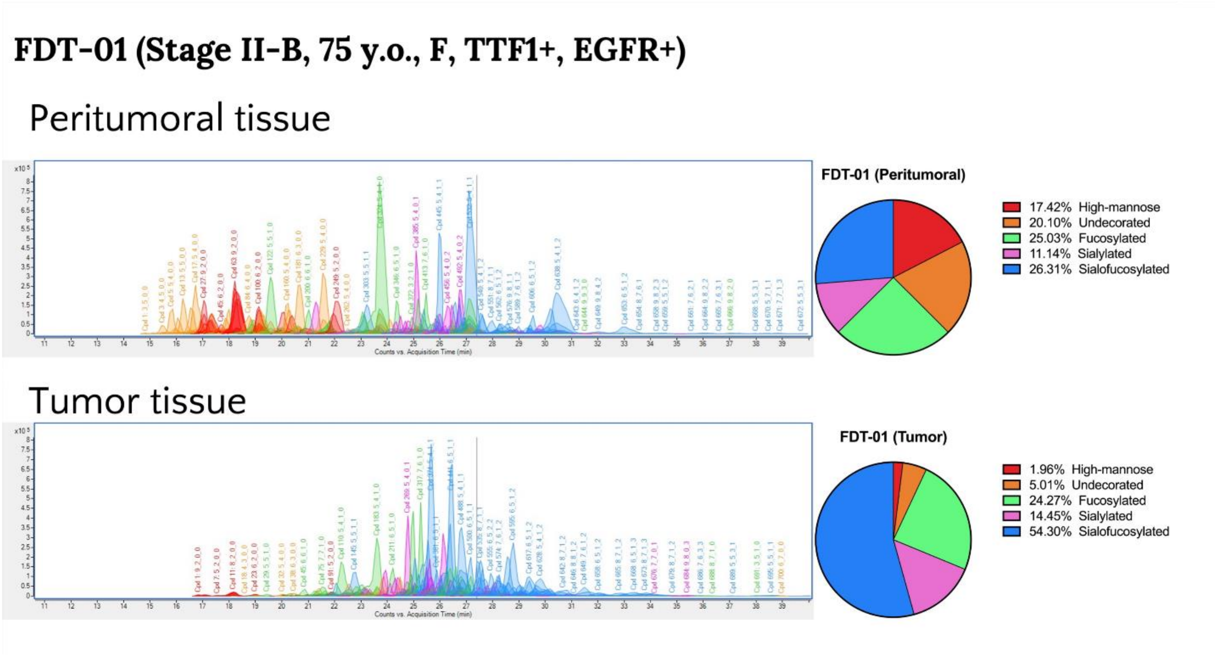

Figure S3. Representative XICs of tumor and peritumoral tissue N-glycome from patient FDT-01.

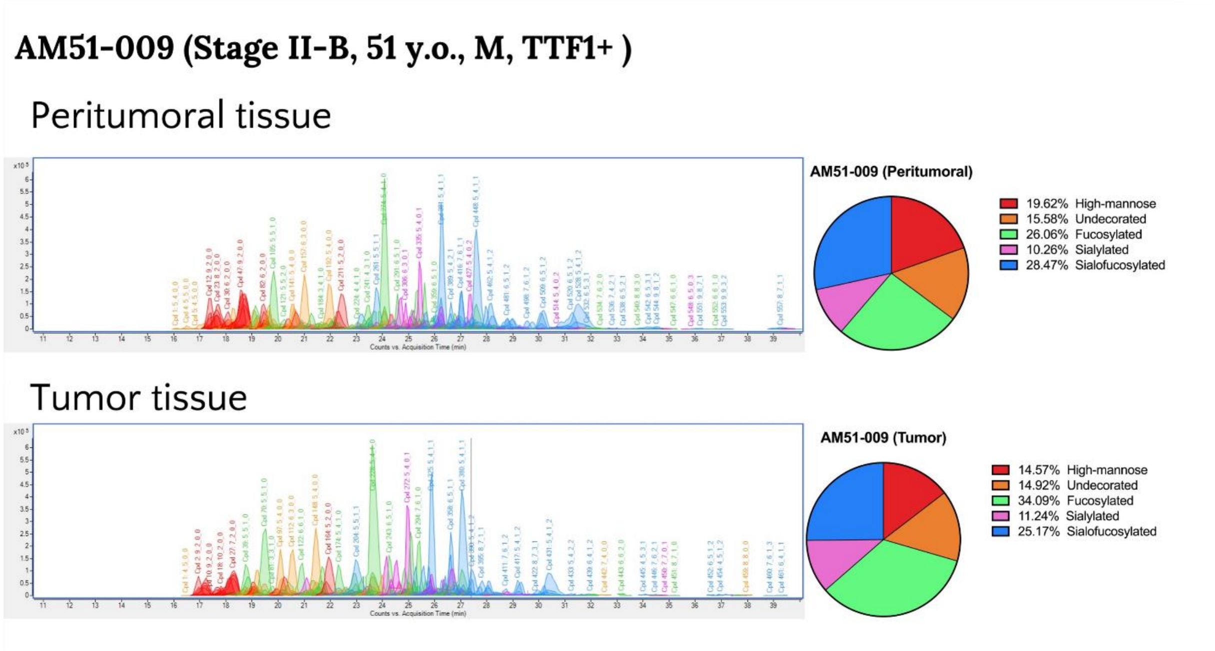

Figure S4. Representative XICs of tumor and peritumoral tissue N-glycome from patient AM51-009.

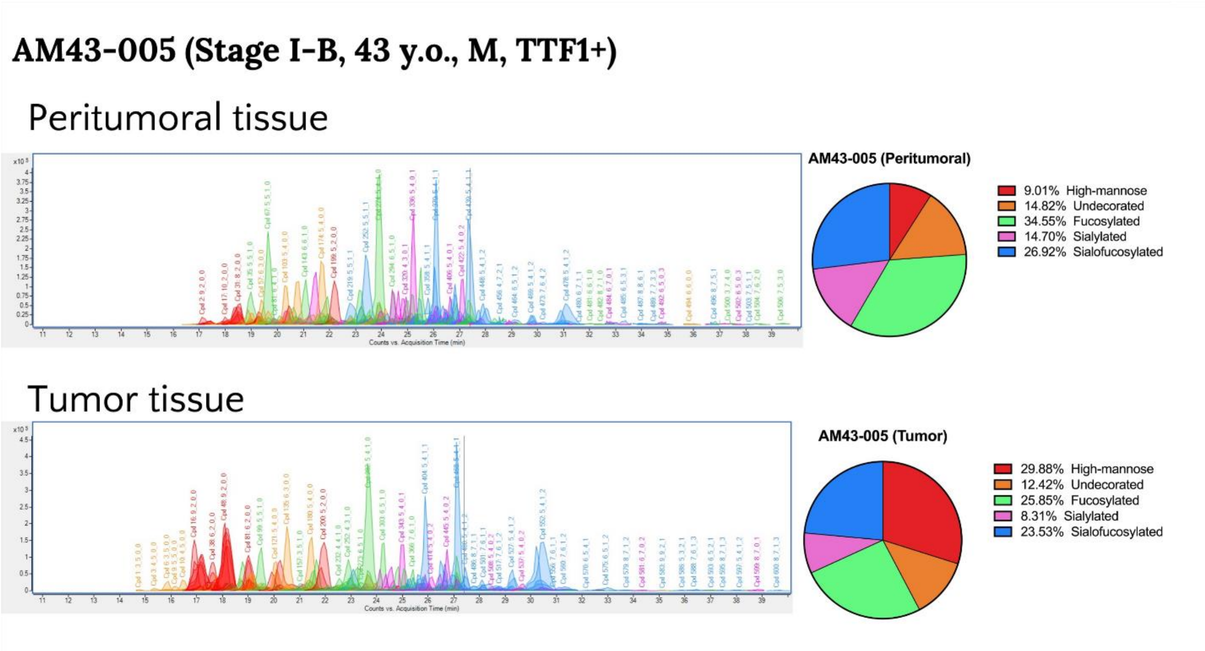

Figure S5. Representative XICs of tumor and peritumoral tissue N-glycome from patient AM43-005.

.

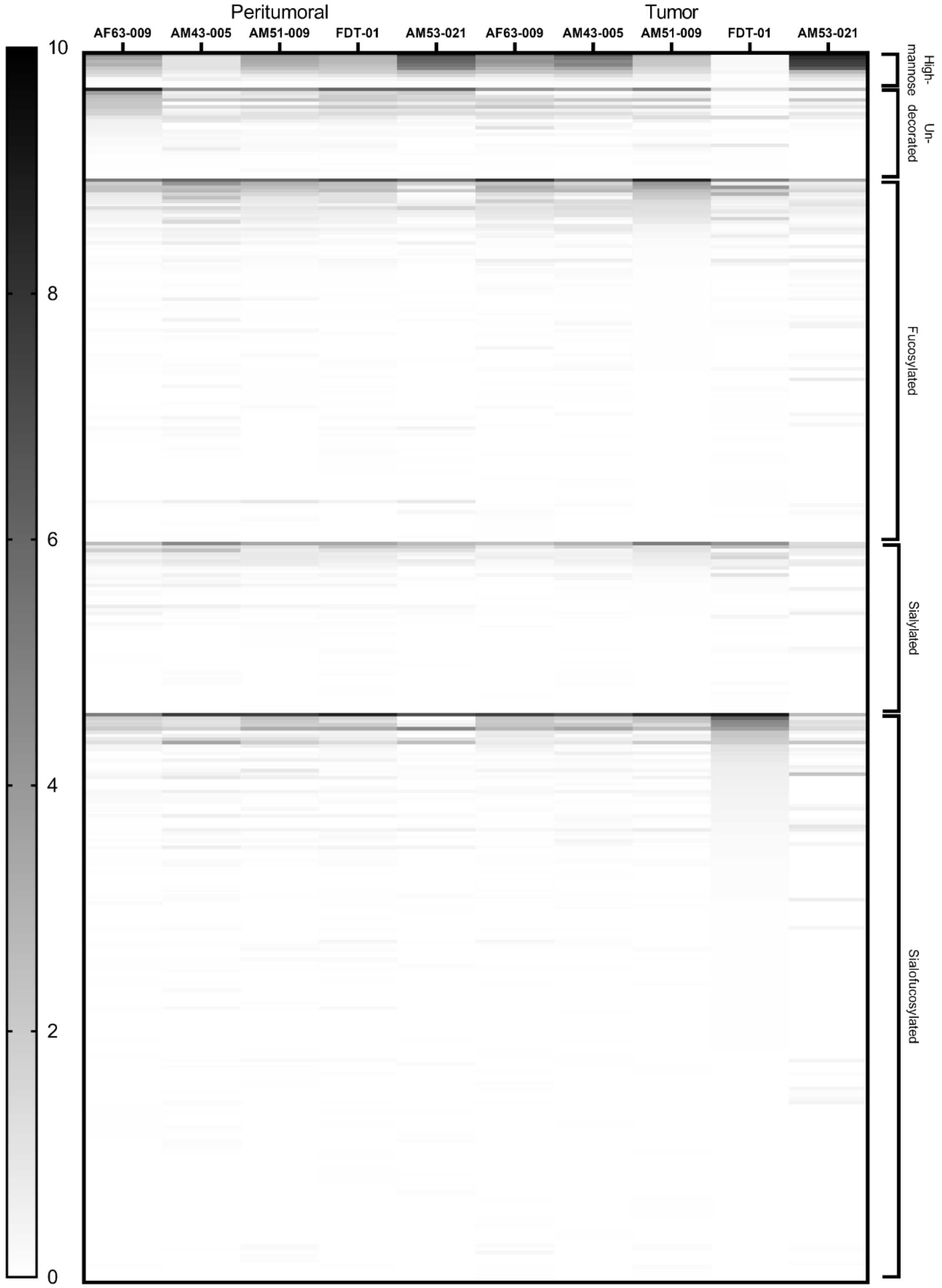

Figure S6. Relative abundance of all N-glycans across all samples.

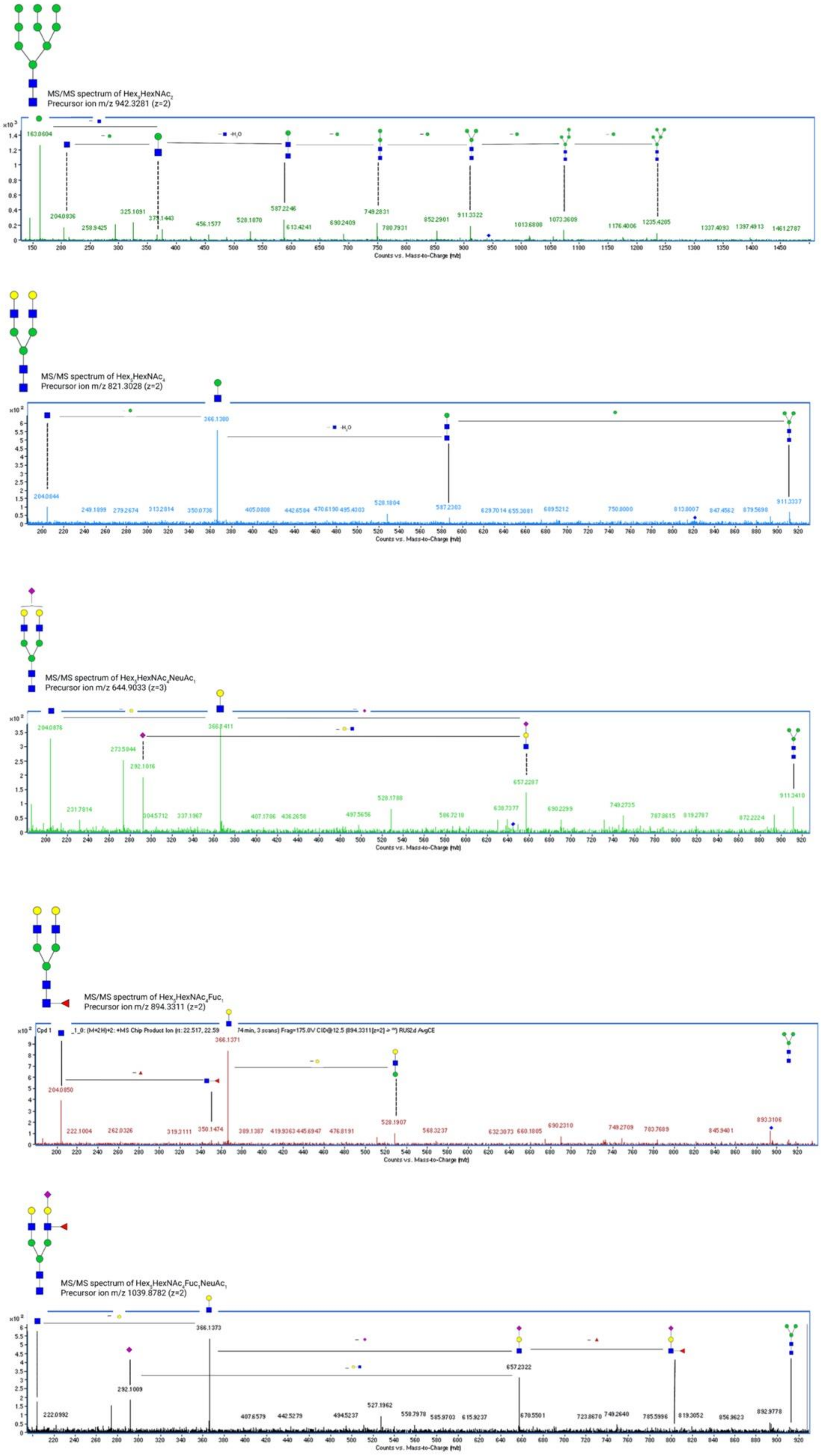

Figure S7. Representative MS/MS spectra of selected N-glycan structures detected in tissue samples.

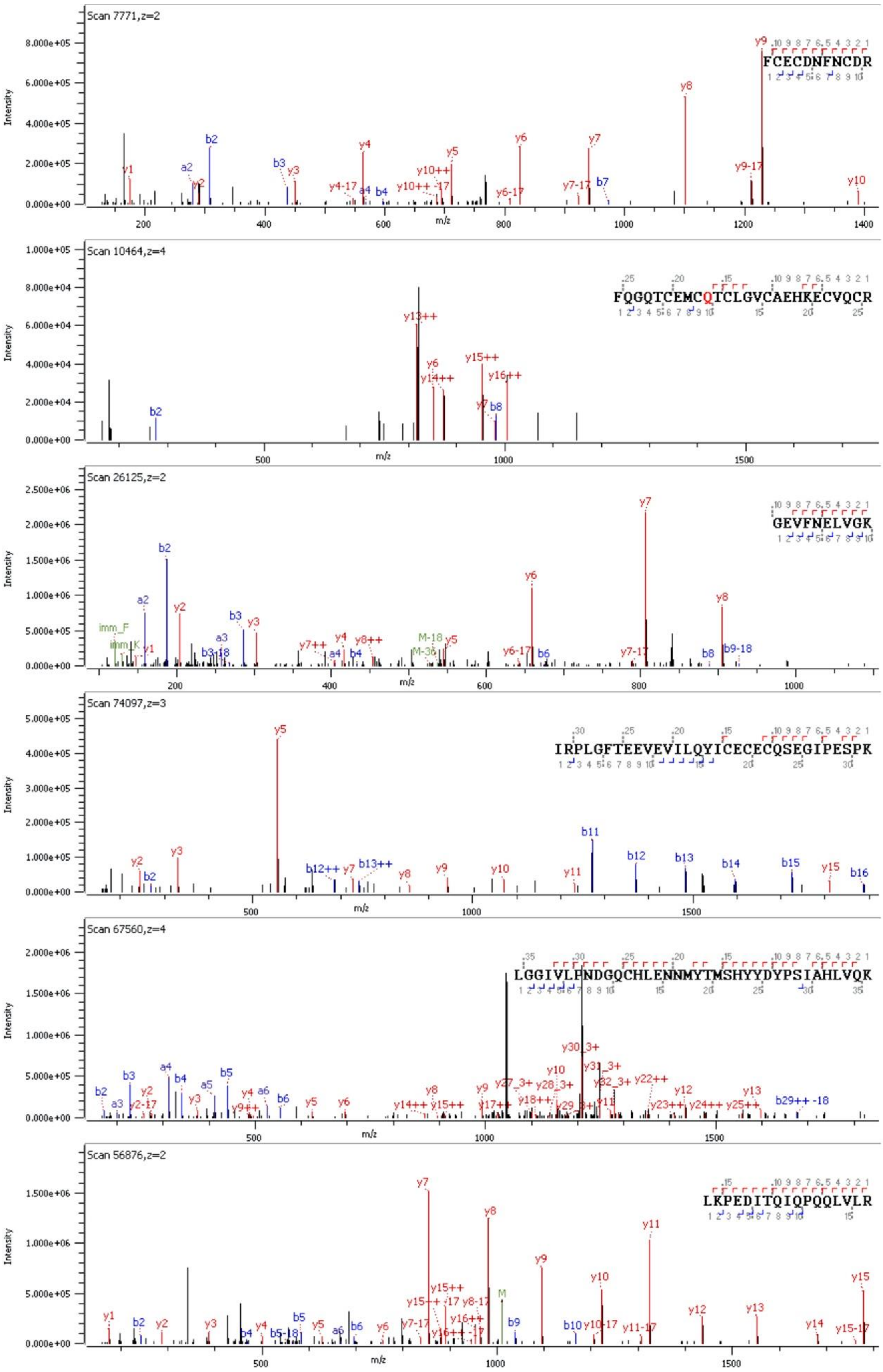
Scan 7771,z=2
FCECDNFNCDR
Scan 10464,z=4
FQGQTCEMCQTCLGVCAEHKECVQCR
Scan 26125,z=2
GEVFNELVGK
Scan 74097,z=3
IRPLGFTEEVEVILQYICECECQSEGIPESPK
Scan 67560,z=4
LGGIVLPNDGQCHLENNMYTMSHYYDYPSIAHLVQK
Scan 56876,z=2
LKPEDITQIQPQQLVLR
Intensity
m/z

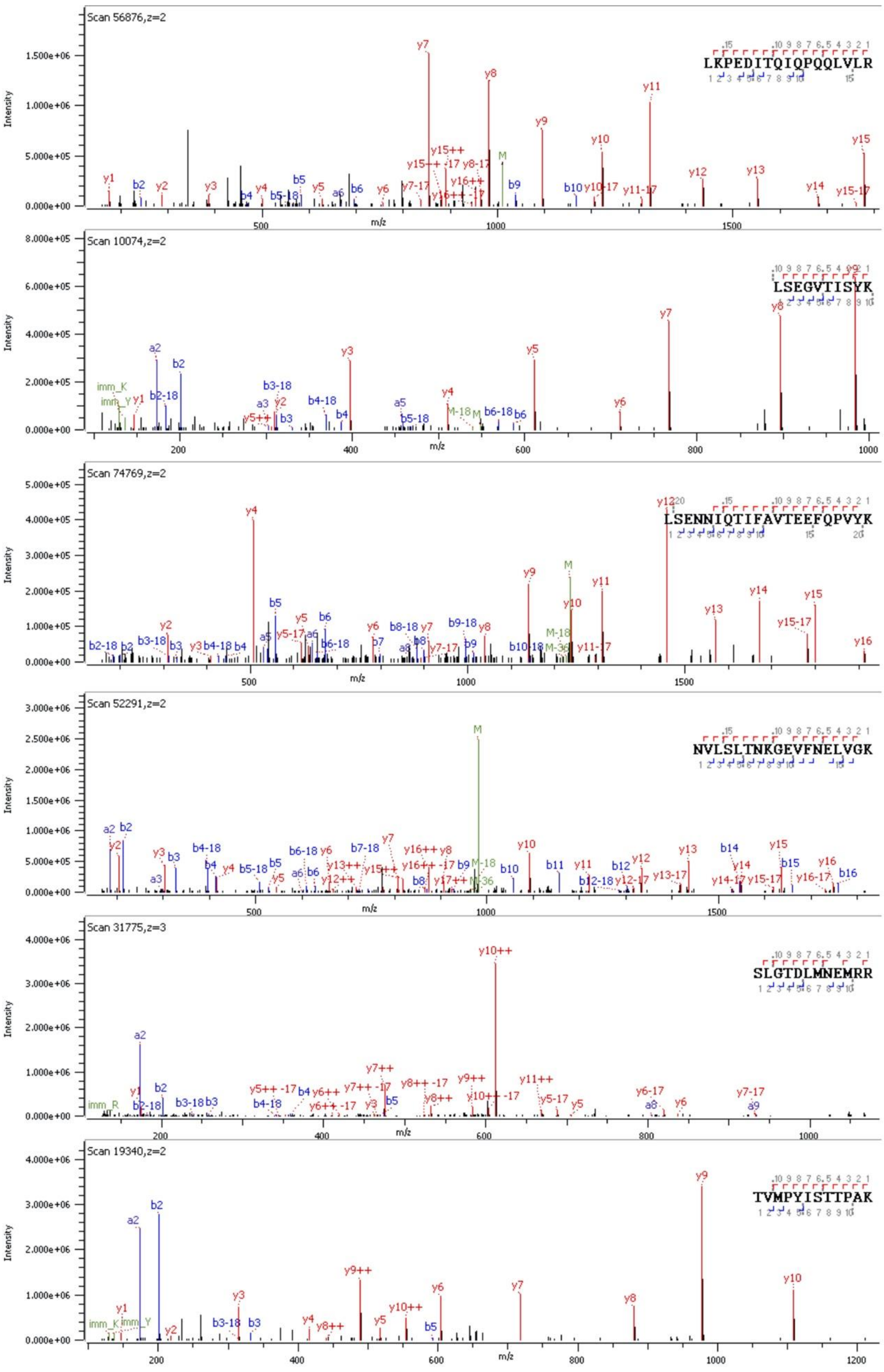
Scan 56876,z=2
LKPEDITQIQPQQLVLR
Scan 10074,z=2
LSEGVTISYK
Scan 74769,z=2
LSENNIQTIFAVTEEFQPVYK
Scan 52291,z=2
NVLSLTNKGEVFNELVGK
Scan 31775,z=3
SLGTDLMNEMRR
Scan 19340,z=2
TVMPYISTTPAK
Intensity
m/z

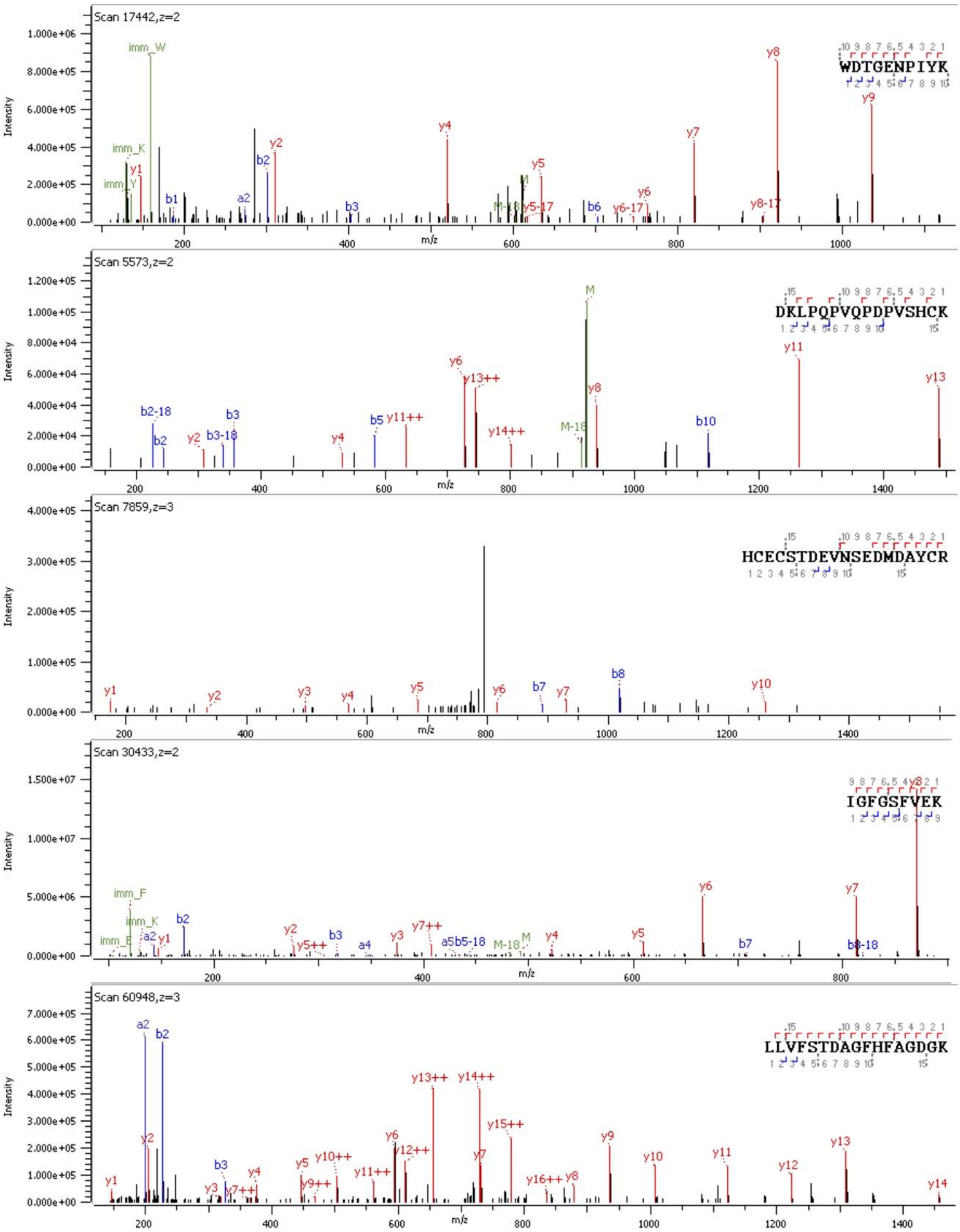

Figure S8. Representative MS/MS spectra of ITGB1 peptides detected in tissue samples.

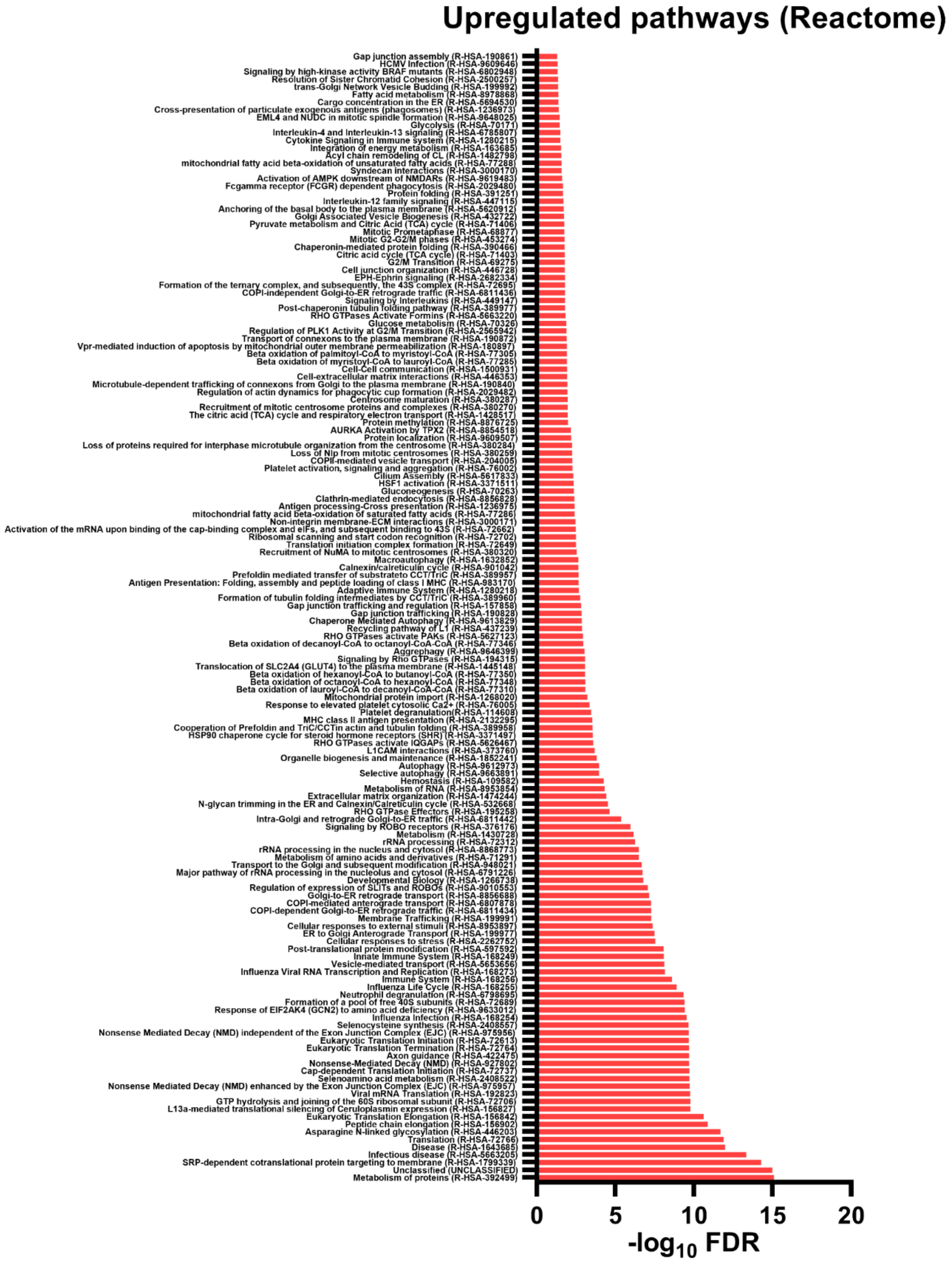

Figure S9. Significantly-enriched Reactome pathways based on significantly-overexpressed proteins in tumor tissues.

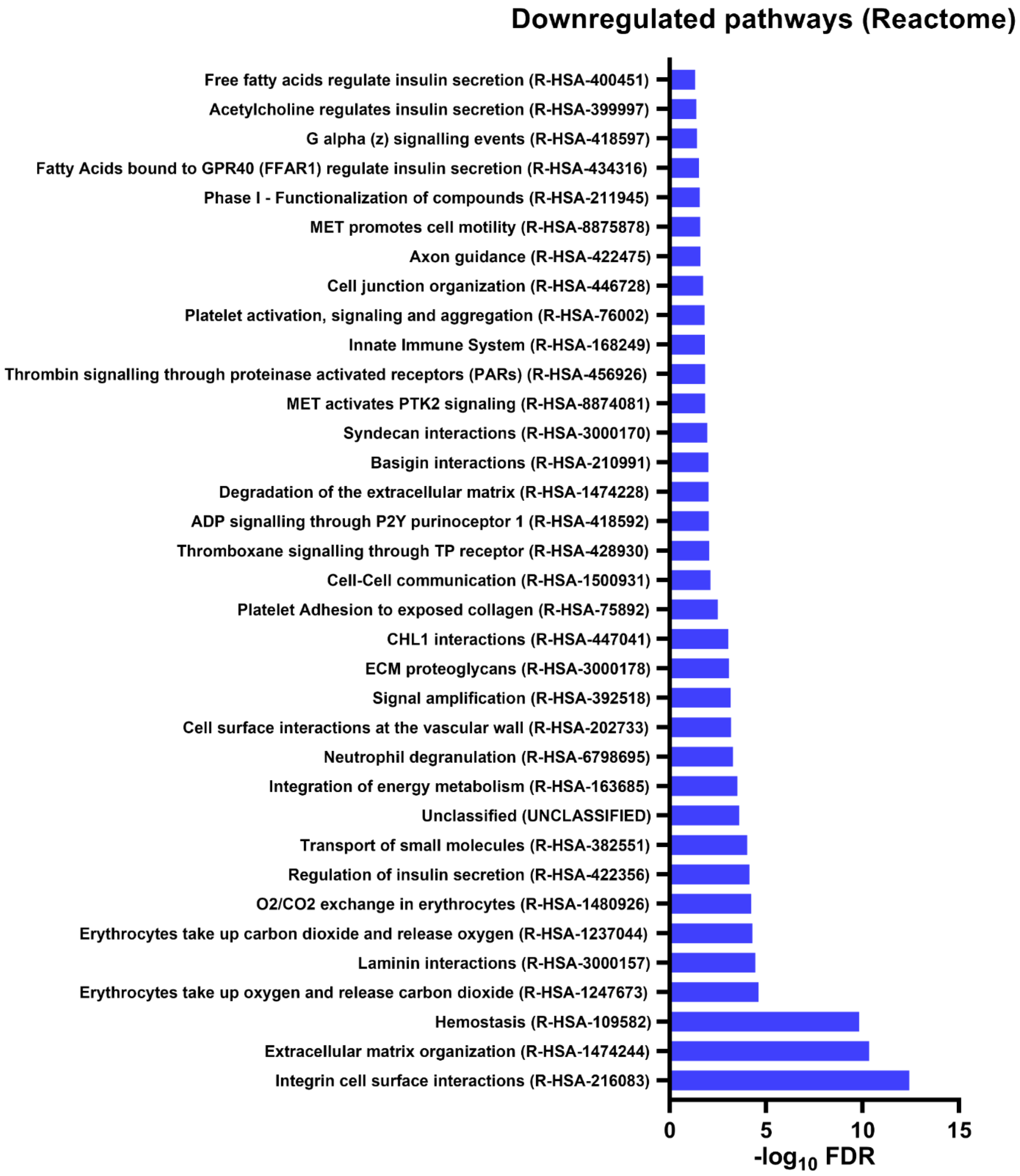

Figure S10. Significantly-enriched Reactome pathways based on significantly-underexpressed proteins in tumor tissues.

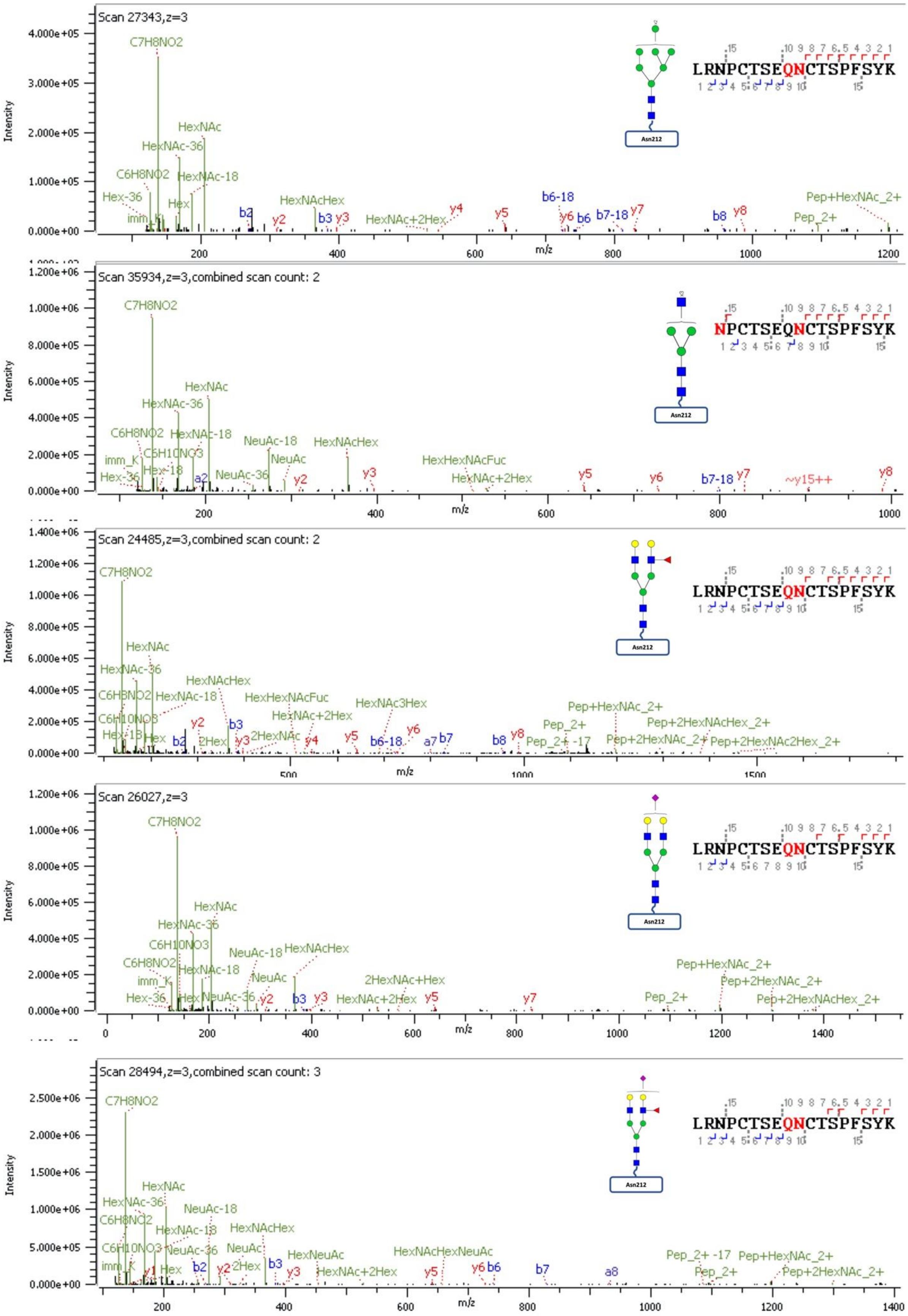

Figure S11. Representative MS/MS spectra of ITGB1 glycopeptides at glycosite Asn212 detected in tissue samples.

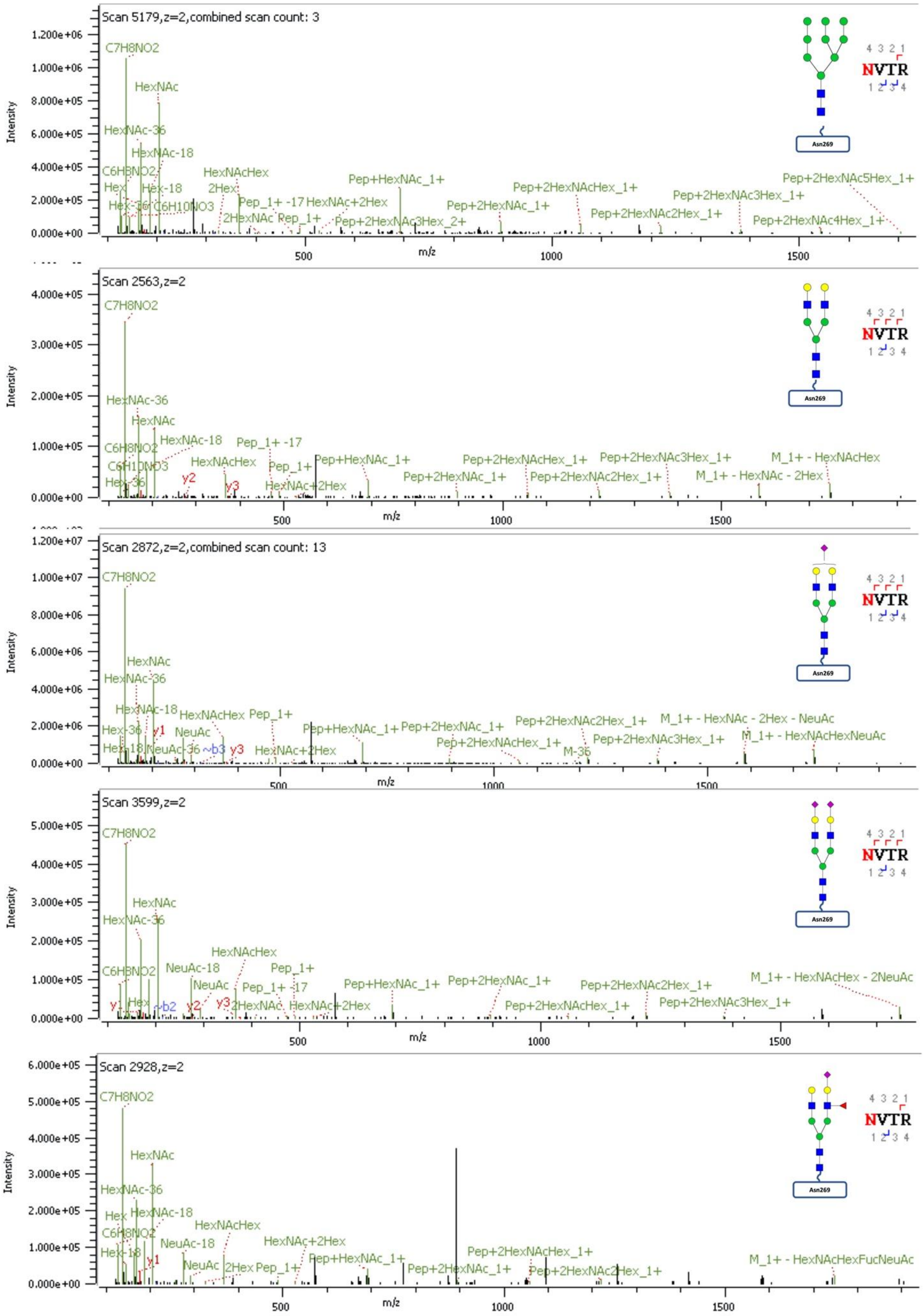

Figure S12. Representative MS/MS spectra of ITGB1 glycopeptides at glycosite Asn269 detected in tissue samples.

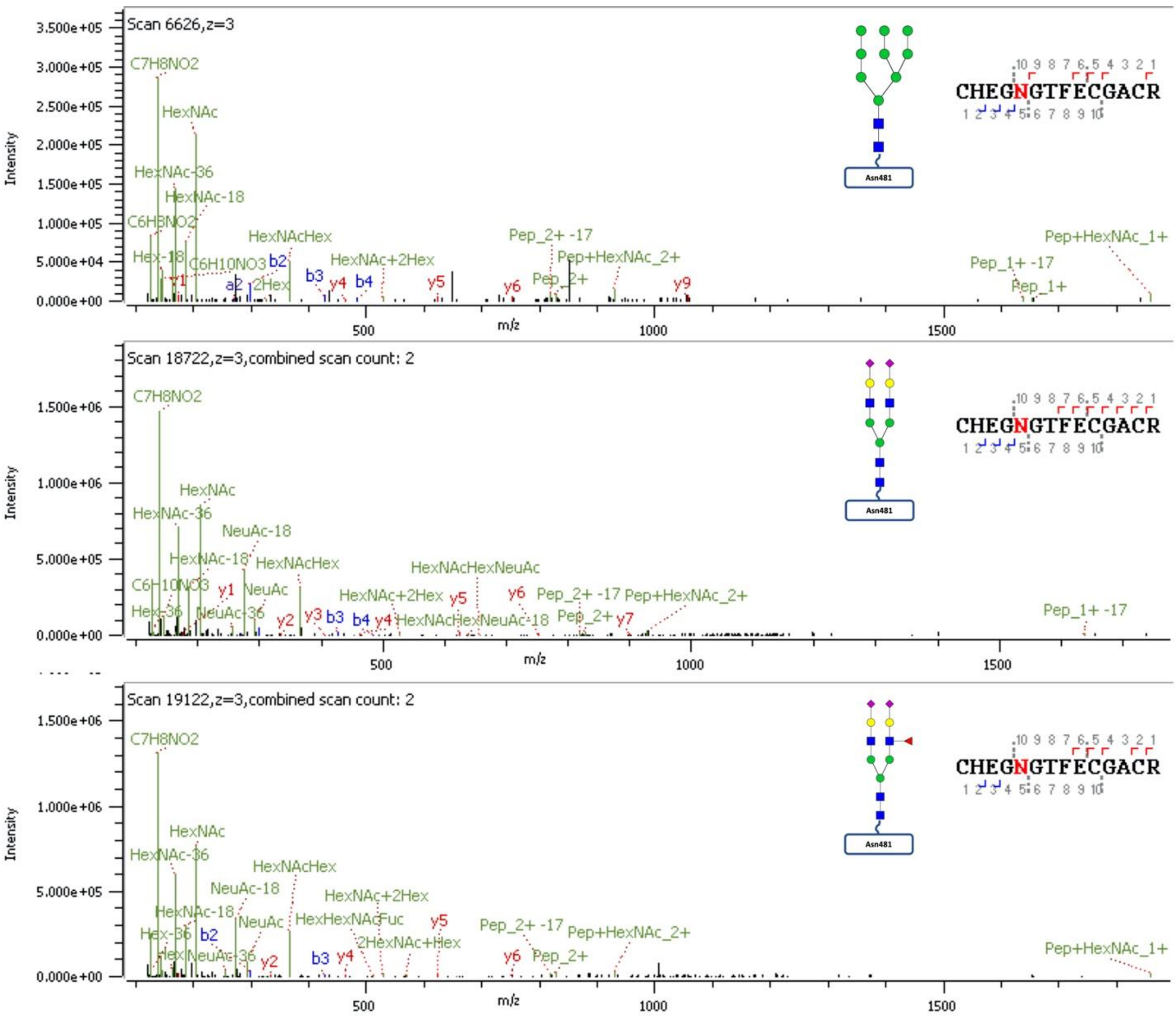

Figure S13. Representative MS/MS spectra of ITGB1 glycopeptides at glycosite Asn481 detected in tissue samples.

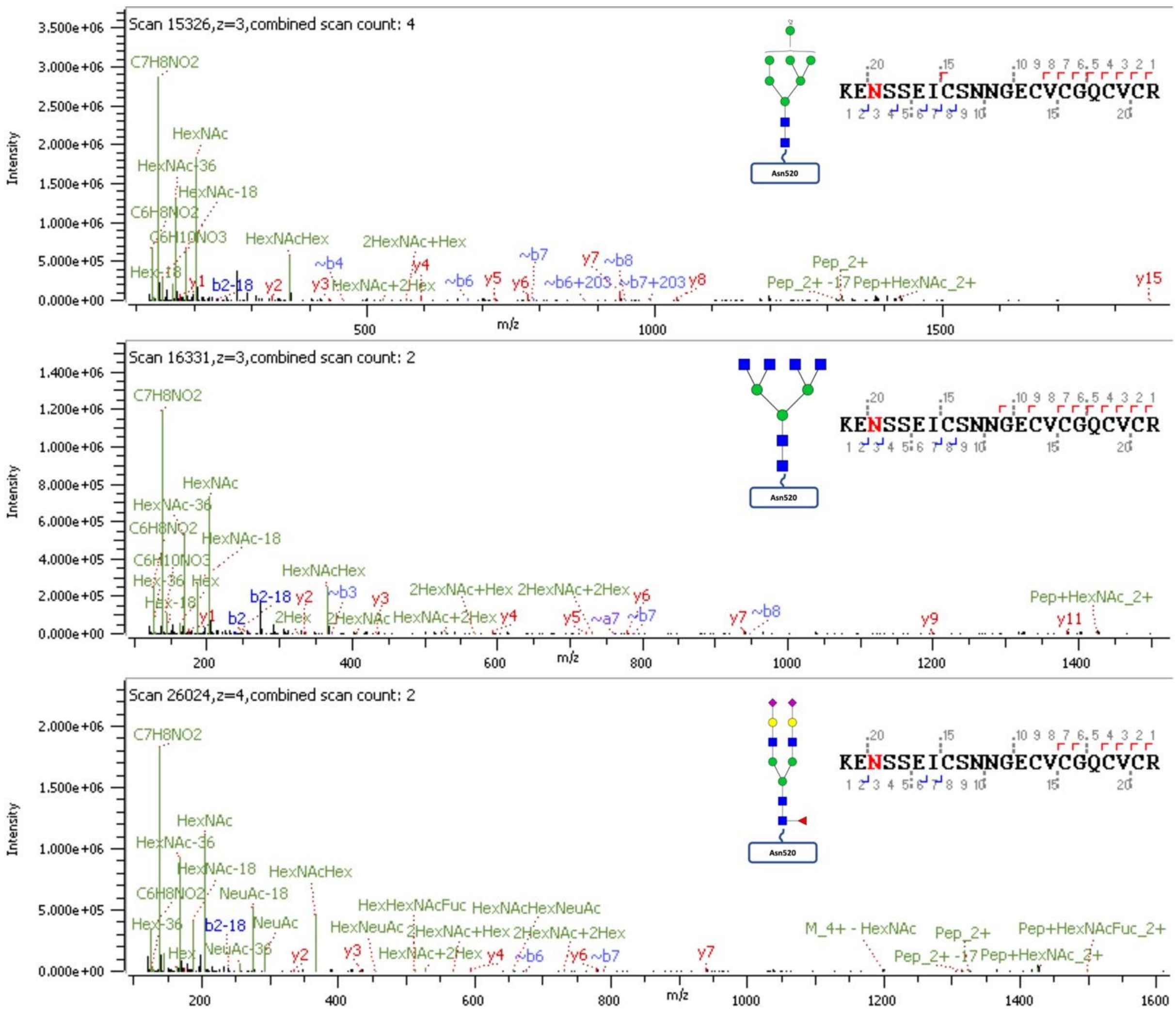

Figure S14. Representative MS/MS spectra of ITGB1 glycopeptides at glycosite Asn520 detected in tissue samples.

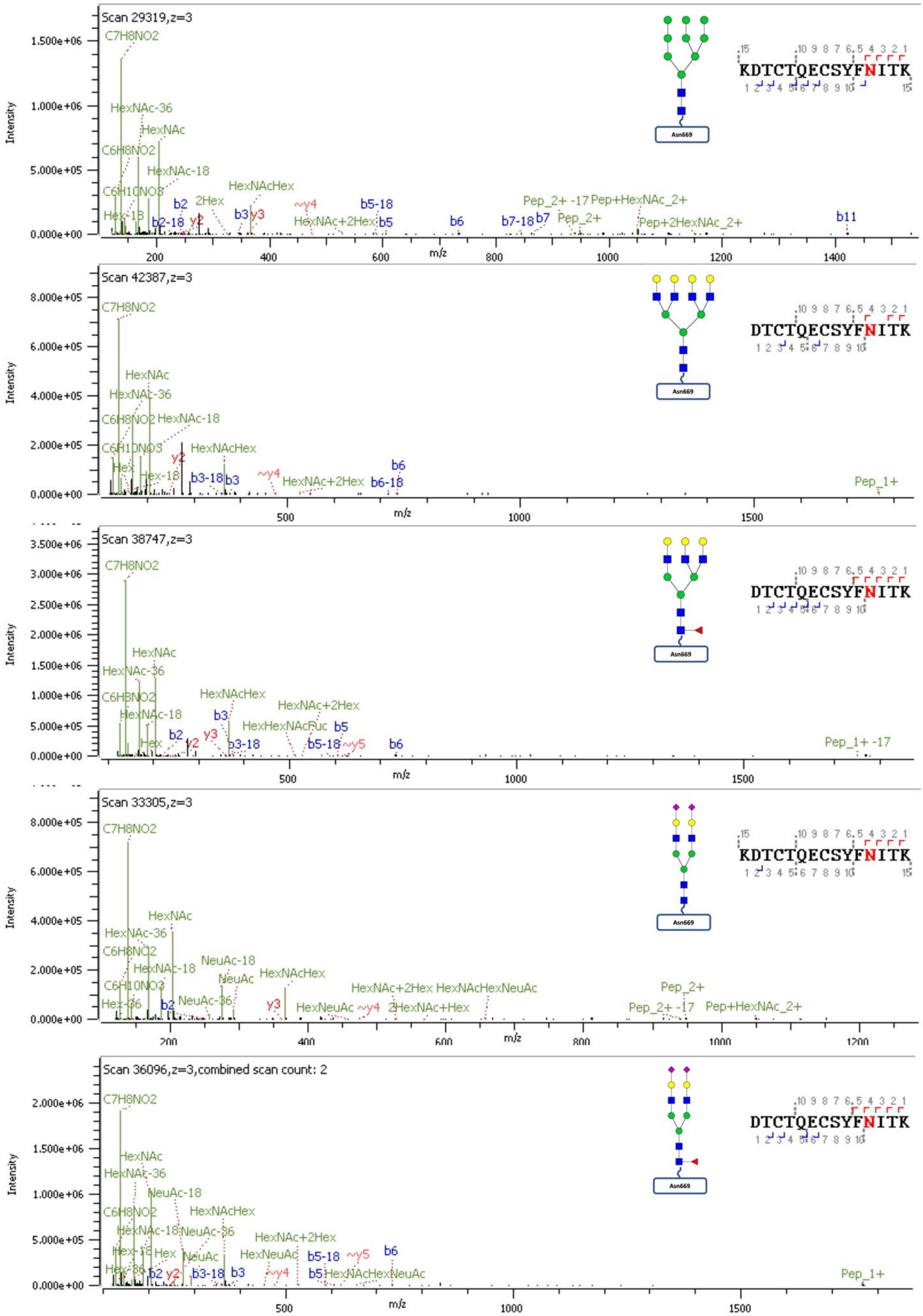

Figure S15. Representative MS/MS spectra of ITGB1 glycopeptides at glycosite Asn669 detected in tissue samples.

Table S1. Inclusion and exclusion criteria.

	Both cases and controls	Patient cases	Controls
Inclusion criteria	• Filipino (up to second degree of consanguinity)<br>• 18-85 years old<br>• Willing to execute an informed consent (IC)<br>• No other serious complicating illness	• Chemotherapy and radiotherapy naïve;<br>• Histopathology confirmed with lung adenocarcinoma and is already set or will be scheduled for surgery as part of their treatment<br>• Histopathology confirmed with lung adenocarcinoma and is already set or will be scheduled for draining of pleural fluid.<br>• Patients with lung mass and will be scheduled for fine needle biopsy.	• Physically assessed to be healthy by a physician (i.e. normal vital signs, physical examination)<br>• Normal chest X-ray result within the last 6 months<br>• No smoking history within the last 2 years<br>• No history of malignancies<br>• Did not have serious illness in the last 6 months
Exclusion criteria	• Patients that are pregnant/suspected pregnant and breastfeeding will be excluded from the study .<br>• Patients diagnosed with pneumonia and TB will also be excluded<br>• Patients with the presence of concomitant autoimmune disease or immunodeficiency as confirmed by viral load testing.<br>• Patients diagnosed with diabetes mellitus will also be excluded as high glucose levels may potentially increase the degree of glycosylation that may be observed in the cells both intracellularly and extracellularly	• Patients that have undergone chemotherapy, radiotherapy and those with concomitant cancer other than lung adenocarcinoma.<br>• Patients that are histopathologically diagnosed with small cell carcinoma, unspecified cell type, malignant cell and non-small cell carcinoma other than adenocarcinoma will be excluded from the study.	
